# The small non-coding RNA RsaE influences extracellular matrix composition in *Staphylococcus epidermidis* biofilm communities

**DOI:** 10.1371/journal.ppat.1007618

**Published:** 2019-03-14

**Authors:** Sonja M. K. Schoenfelder, Claudia Lange, Srinivasa Abishek Prakash, Gabriella Marincola, Maike F. Lerch, Freya D. R. Wencker, Konrad U. Förstner, Cynthia M. Sharma, Wilma Ziebuhr

**Affiliations:** University of Würzburg, Institute of Molecular Infection Biology, Würzburg, Germany; National Institutes of Health, UNITED STATES

## Abstract

RsaE is a conserved small regulatory RNA (sRNA) which was previously reported to represent a riboregulator of central carbon flow and other metabolic pathways in *Staphylococcus aureus* and *Bacillus subtilis*. Here we show that RsaE contributes to extracellular (e)DNA release and biofilm-matrix switching towards polysaccharide intercellular adhesin (PIA) production in a hypervariable *Staphylococcus epidermidis* isolate. Transcriptome analysis through differential RNA sequencing (dRNA-seq) in combination with confocal laser scanning microscopy (CLSM) and reporter gene fusions demonstrate that *S*. *epidermidis* protein- and PIA-biofilm matrix producers differ with respect to RsaE and metabolic gene expression. RsaE is spatiotemporally expressed within *S*. *epidermidis* PIA-mediated biofilms, and its overexpression triggers a PIA biofilm phenotype as well as eDNA release in an *S*. *epidermidis* protein biofilm matrix-producing strain background. dRNA-seq and Northern blot analyses revealed RsaE to exist as a major full-length 100-nt transcript and a minor processed species lacking approximately 20 nucleotides at the 5'-end. RsaE processing results in expansion of the mRNA target spectrum. Thus, full-length RsaE interacts with *S*. *epidermidis* antiholin-encoding *lrgA* mRNA, facilitating bacterial lysis and eDNA release. Processed RsaE, however, interacts with the 5'-UTR of *icaR* and *sucCD* mRNAs, encoding the *icaADBC* biofilm operon repressor IcaR and succinyl-CoA synthetase of the tricarboxylic acid (TCA) cycle, respectively. RsaE augments PIA-mediated biofilm matrix production, most likely through activation of *icaADBC* operon expression via repression of *icaR* as well as by TCA cycle inhibition and re-programming of staphylococcal central carbon metabolism towards PIA precursor synthesis. Additionally, RsaE supports biofilm formation by mediating the release of eDNA as stabilizing biofilm matrix component. As RsaE itself is heterogeneously expressed within biofilms, we consider this sRNA to function as a factor favoring phenotypic heterogeneity and supporting division of labor in *S*. *epidermidis* biofilm communities.

## Author summary

Bacterial biofilms are highly organized structures which functionally emulate multicellular organisms, last but not least through heterogeneous gene expression patterns displayed by biofilm subpopulations. Here we analyzed the functions of the non-coding RNA RsaE in *Staphylococcus epidermidis* biofilm communities. RsaE exerted unexpected influences on *S*. *epidermidis* biofilm matrix composition by triggering localized eDNA release and facilitating PIA expression. RsaE accomplishes these effects by targeting mRNAs involved in bacterial lysis control, *icaADBC* expression and TCA cycle activity, with RsaE undergoing processing to exploit its full target potential. Interestingly, RsaE interaction with lysis-engaged *lrgA* mRNA is specific for *S*. *epidermidis lrgA*, but does not occur with *lrgA* mRNA from *S*. *aureus*, suggesting species-specific differences in staphylococcal lysis control. We speculate that RsaE-mediated bacterial lysis might represent a form of bacterial altruism contributing to biofilm structuring by providing nutrients to neighboring bacterial cells as well as by releasing eDNA as stabilizing biofilm matrix component. Due to its heterogeneous expression, we consider RsaE as a supporting factor that facilitates population diversity. Together, the data give insight into an unanticipated role of sRNAs as players in *S*. *epidermidis* biofilm organization.

## Introduction

Life in biofilms is a widespread phenomenon in the bacterial world. Many planktonically living bacteria are capable of aggregating on surfaces and to each other by encasing in a self-produced extracellular matrix, making biofilm formation a common and in some species even the preferred bacterial lifestyle. Typically, biofilm-associated bacteria display pronounced heterogenous gene expression patterns, a feature which is very similar to multicellular organisms [1, 2]. The mechanisms underlying the generation and maintenance of diversity in biofilms and, more general, in bacterial communities, seem to be manifold. They may involve regulatory pathways (including quorum-sensing circuits), reversible and non-reversible genetic events, but also stochastic variations during transcription and translation, resulting in fluctuations of protein levels [3]. Here we focus on factors influencing heterogeneity in the prototype biofilm-forming bacterium and opportunistic nosocomial pathogen *Staphylococcus epidermidis* [4]. Biofilm formation of *S*. *epidermidis* typically occurs on the inert surfaces of medical devices and involves the production of a self-produced extracellular matrix [5]. In *S*. *epidermidis*, the biofilm matrix may consist of the polysaccharide intercellular adhesin (PIA), which is a beta-1,6 linked *N-*acetylglucosamine homopolymer whose synthesis is mediated by the enzymes encoded by the *icaADBC* operon [6]. Alternatively, *S*. *epidermidis* can produce biofilms independent of PIA for example through expression of the accumulation associated protein Aap and other cell surface associated proteins, forming a protein-based biofilm matrix [7, 8]. In addition, extracellular DNA (eDNA) forms a significant component of the biofilm matrix as well [9]. In *S*. *epidermidis*, biofilm formation is a versatile feature undergoing extensive phenotypic variation. Thus, biofilm-negative variants arise regularly from a biofilm-forming population, through both reversible phase variation events and through irreversible deletions and rearrangements of DNA fragments carrying biofilm-associated genes [10–13]. Finally, the bacterium was demonstrated to switch spontaneously between protein- and PIA-mediated biofilm matrix production (and *vice versa*), suggesting a high degree of phenotypic heterogeneity within *S*. *epidermidis* biofilm communities [7, 8, 10, 14–16]. In this report, we study biofilm matrix heterogeneity using a hypervariable clinical *S*. *epidermidis* strain as a model organism. The strain, which was previously obtained from blood cultures and cerebrospinal fluid during a severe and fatal *S*. *epidermidis* infection, constantly generates variants differing in biofilm matrix production [17]. These variants comprise weak protein-mediated biofilm producers (named PS2), originally recovered in the early, and strong PIA-mediated biofilm producers (named PS10) obtained at the later stages of the infection. Also, spontaneous chromosomal deletion mutants, lacking the PIA-mediating *icaADBC* genes, occur regularly in this strain [17].

In order to identify factors that influence heterogeneity in *S*. *epidermidis*, we analyzed in this report gene expression patterns of protein- and PIA-biofilm matrix producers by differential RNA sequencing (dRNA-seq). Amongst many other genes and pathways, we found the conserved staphylococcal small non-coding RNA (sRNA) RsaE to be differentially expressed in *S*. *epidermidis* biofilms. RsaE, which was originally identified in *S*. *aureus*, was shown to act as a riboregulator of central carbon and amino acid metabolism in this organism [18–20]. Here, we analyze the molecular function of RsaE in *S*. *epidermidis* and demonstrate that this sRNA contributes to heterogeneity in *S*. *epidermidis* biofilm communities by supporting PIA-biofilm matrix production as well as by mediating localized bacterial lysis and eDNA release.

## Results

### Biofilm matrix variants display varying carbon flux gene expression patterns

**Fig 1:**
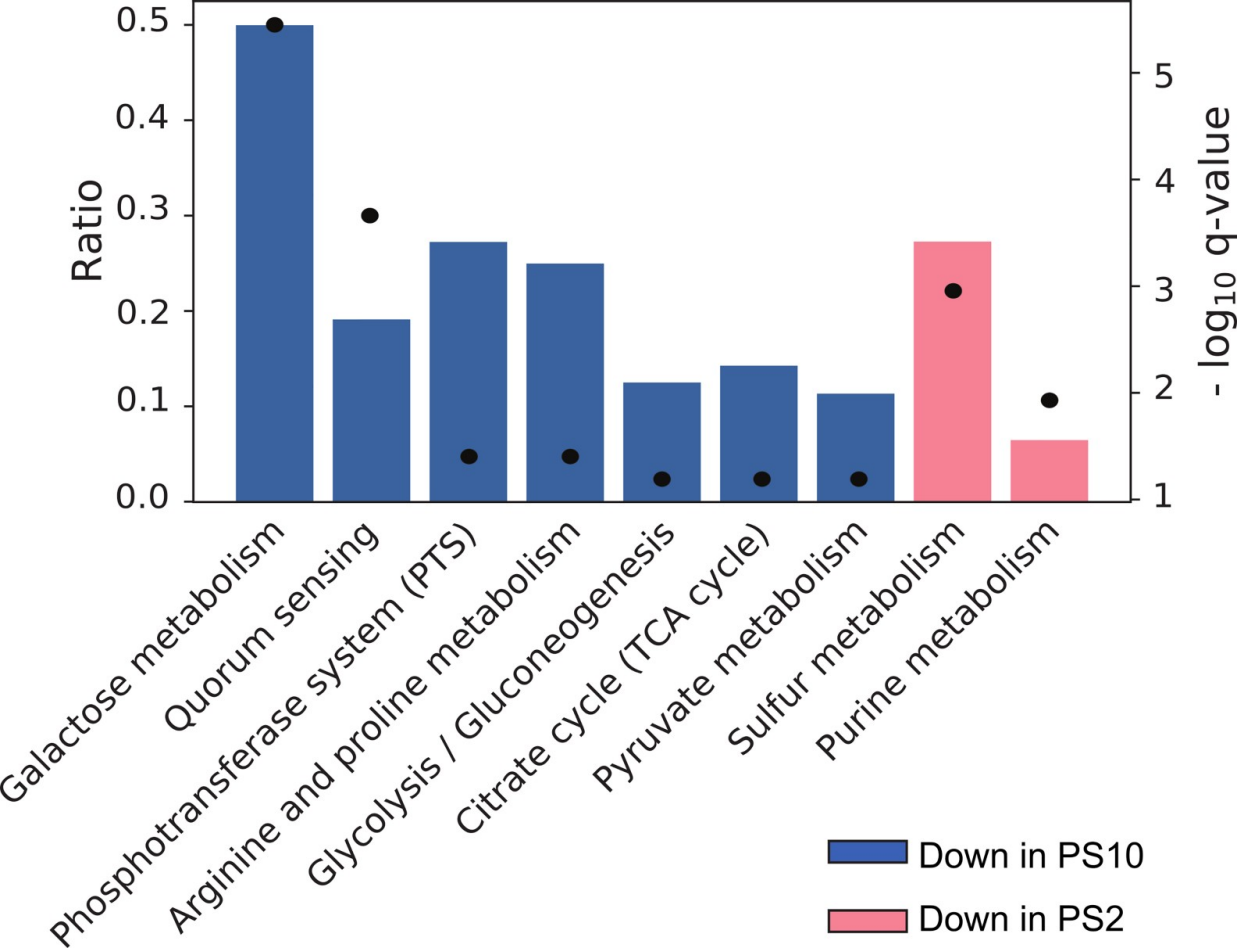
Enrichment of gene ontology (GO) terms among differentially expressed genes between *S*. *epidermidis* PS2 and PS10. The analysis considers differentially expressed genes in the TEX-untreated library of the dRNA-seq experiment with an absolute log_2_(fold-change) ≥ 1 (p-value < 0.05). Bars display the ratio of the number of differentially expressed genes in relation to the total number of genes associated to the respective GO term, while dots represent -log_10_ q-values.

To get an insight into the molecular basis of varying biofilm matrix production in *S*. *epidermidis*, we analyzed the transcription profiles of the PS2 variant (protein biofilm matrix) in comparison to the PS10 variant (PIA biofilm matrix) during growth in batch cultures by dRNA-seq. In the exponential growth stage, we identified 77 genes as differentially expressed (absolute log_2_fold-change ≥1, p<0.05) in the two variants (S1 Table). By performing a pathway enrichment analysis, the majority of these genes were assigned to a number of distinct metabolic pathways and cellular processes (Fig 1). Interactive visualization of the complete data set is shown in S1 Fig. In the PIA producing PS10 variant, transcription of genes of galactose and pyruvate metabolism, glycolysis/ gluconeogenesis and the tricarboxylic acid (TCA) cycle were downregulated. Except for a glucose-specific phosphotransferase (PTS) system, other sugar uptake PTS were repressed in PS10, along with genes involved in arginine and proline metabolism. Other striking features were the upregulation of genes involved in sulfur metabolism and purine synthesis, the latter suggesting induced nucleotide metabolism and DNA synthesis in the PS10 variant (Fig 1). Further, downregulation of the antiholin-encoding *lrgAB* operon was recorded in PS10 along with a diminished expression of the *agr*-quorum-sensing system, including its effector molecule RNAIII (S1 Table). Interestingly, differential expression of a putative novel long non-coding RNA (lncRNA) in the vicinity of the *ica* locus (whose gene is not annotated yet in *S*. *epidermidis* genomes) was recorded as well. This lncRNA, named IcaZ, is indispensable for PIA-mediated production and its characterization will be described in detail in a separate study. Taken together, the transcription profile is in good agreement with a status that would enable PIA synthesis in PS10 by redirecting carbon sources from energy gain through the TCA cycle towards aminosugar and eventually PIA precursor synthesis [21].

### Biofilm matrix variants differ with respect to riboregulator RsaE expression

**Fig 2:**
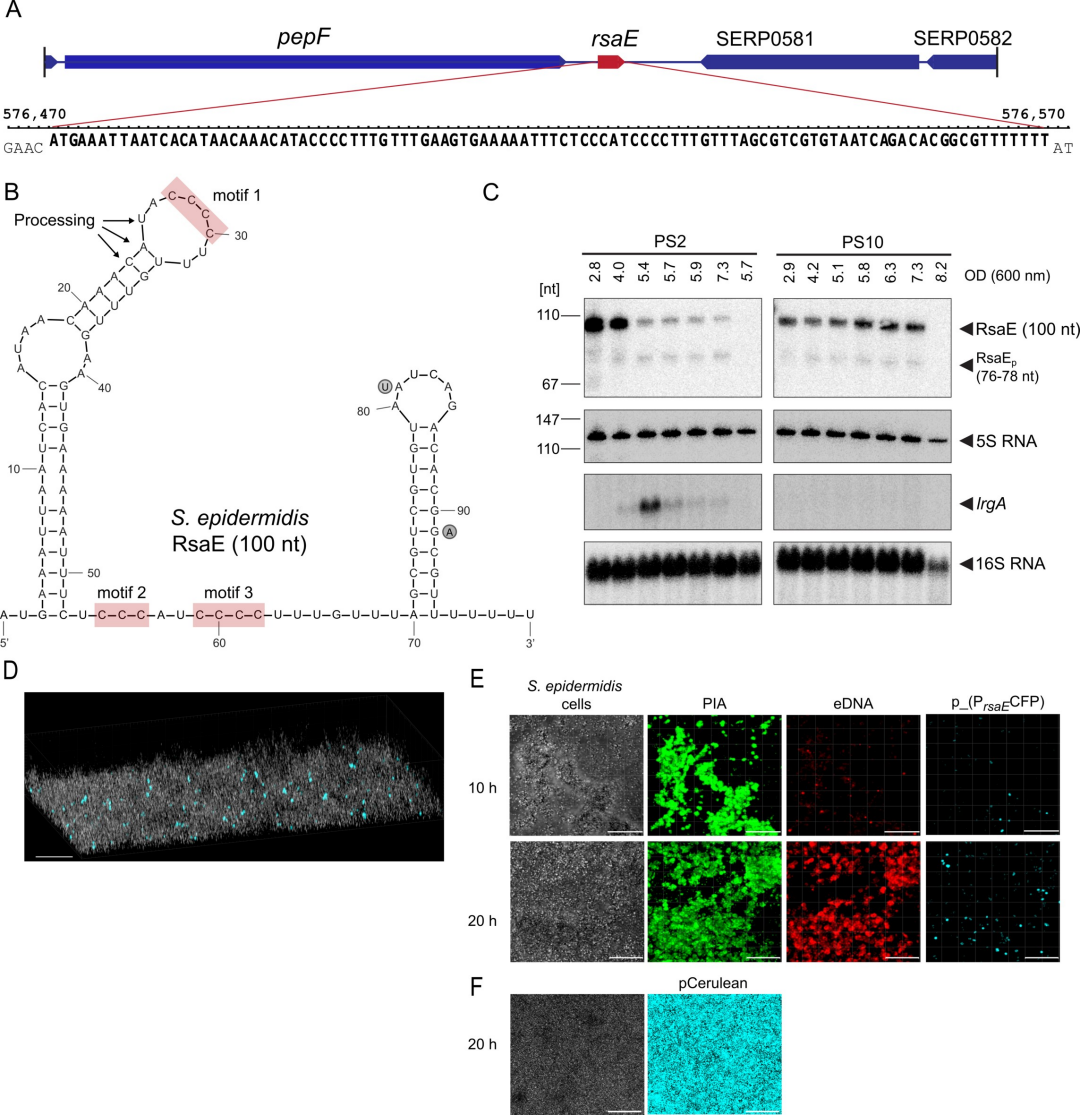
Genetic localization, structure and expression of RsaE in *S*. *epidermidis*. (*A*) Genetic localization (position 576.475 to 576.574) and nucleotide sequence of the *rsaE* gene in the *S*. *epidermidis* RP62A reference genome (NC_002976). (*B*) Secondary structure of RsaE. Arrows mark putative processing sites of RsaE (see text for details) and red boxes highlight unpaired C-rich motifs prone to mediate mRNA binding [19]. Circled nucleotides next to the sequence indicate positions of two varying nucleotides present in *S*. *aureus* RsaE. (*C*) Northern blot detection of *rsaE* and *lrgA* expression during growth of *S*. *epidermidis* PS2 and PS10 in batch cultures at 37°C in TSB. Optical densities (OD_600_) at which samples were taken are indicated, with the last samples taken after 24 hours. Arrowheads mark full-length and processed (RsaE_p_) RsaE species, *lrgA* transcript as well as 5S and 16S rRNA used as loading controls. (*D*) 3D-CLSM image of a *S*. *epidermidis* PS10 biofilm population (grey cells) grown in chamber slides for 20 hours. The strain carries plasmid p_(P_*rsaE*_CFP) in which the *rsaE* promoter is fused to the blue-fluorescent Cerulean protein gene *cfp* as reporter. Light blue cells represent bacteria with an active *rsaE* promoter. (*E*) CLSM images of an *S*. *epidermidis* PS10 p_(P_*rsaE*_CFP) biofilm during growth (10 and 20 hours) in chamber slides. The total bacterial cell mass is visualized by transmission microscopy (left panels), PIA matrix is stained in green by Alexa Fluor 488 wheat-germ agglutinin (middle left), and eDNA (in red) by Ethidium Homodimer III (middle right). Cerulean expression (light blue) highlights *rsaE*-expressing bacterial cells (right panels). *(F)* CLSM images of *S*. *epidermidis* PS10 carrying control plasmid pCerulean in which *cfp* is under control of an ATc-inducible promoter. Images were taken after 20 hours of growth in chamber slides in TSB supplemented with 100 ng/ ml ATc. The total bacterial cell mass is visualized by transmission microscopy (left panels), Cerulean expression (light blue) highlights CFP expressing bacterial cells (right panels). Bars represent 20 μm.

Differential expression of central carbon flux genes, including TCA cycle genes, was the most striking result of the transcription profiling experiment. In the light of previous findings indicating that (i) in *S*. *aureus* TCA cycle activity is influenced by the sRNA RsaE [18], and that (ii) in *S*. *epidermidis* an impaired TCA cycle favours PIA synthesis [21], we set out to investigate whether or not RsaE might play a role in the switch of biofilm matrix production in the *S*. *epidermidis* PS strains. By bioinformatic means, RsaE was predicted previously to be conserved among the Bacillales [18, 22]. Fig 2A depicts the putative genetic localization of the *rsaE* gene in an intergenic region between *pepF* (SERP0580) and SERP0581 of the *S*. *epidermidis* RP62A reference genome. Inspection of this region in the dRNA-seq data set identified a transcript of the corresponding size (100 nucleotides) and orientation, indicating that RsaE is expressed in *S*. *epidermidis*, with variant PS10 displaying 2.5-fold higher transcription levels than PS2 (Fig 2C; Fig 7A). *S*. *epidermidis* RsaE is likely to adopt an identical structure as previously experimentally shown for RsaE of *S*. *aureus*, as the primary sequences are highly conserved and differ only by two nucleotides towards the 3'-end, and which do not involve the C-stretches mediating target mRNA-binding [19, 22, 23] (Fig 2B). To study RsaE expression in the PS2 and PS10 variants in more detail, Northern blot analyses were performed during growth in liquid culture. *rsaE*-specific hybridization of total RNA obtained during exponential as well as in the stationary growth stage revealed that RsaE is expressed by both variants, but with strikingly different expression patterns over time (Fig 2C). In PS2, RsaE was highly expressed during early- and mid- exponential growth and decreased in the late exponential stage (Fig 2C). In contrast, PS10 exhibited high RsaE transcription levels during the entire exponential growth phase. In the stationary stage, RsaE transcription was no longer detectable in both variants (Fig 2C). Also in both variants, a faint additional RsaE-specific band was detectable (RsaE_p_ in Fig 2C) which was smaller than the main transcript (see below for details).

### RsaE is spatiotemporally expressed within *S*. *epidermidis* PIA-mediated biofilms

Typically, within the multilayered structure of a biofilm, access to nutrients, water and oxygen may vary significantly, resulting in the formation of microniches exhibiting distinct metabolic conditions and gene expression patterns to meet these metabolic challenges. As RsaE is supposed to influence a multitude of metabolic functions, this sRNA is a promising candidate to play a role in biofilm organization. The dRNA-seq and Northern analyses from batch cultures described above detect bulk RNA amounts of transcripts, but do not provide information on the spatiotemporal expression of distinct factors (such as RsaE) within the biofilm architecture. In the PIA-producing PS10 isolate, RsaE transcripts were highly abundant (Fig 2C), and we wondered if all bacteria in an *in situ* biofilm community homogeneously activate RsaE transcription during PIA-mediated biofilm formation. Thus, to study spatiotemporal *rsaE* expression in PIA biofilms, we fused the *rsaE* promoter region to the blue-fluorescent cerulean protein gene *cfp* on a plasmid and transformed the construct into the PS10 isolate. Using cerulean expression as a reporter, we monitored *rsaE* expression by CLSM in *S*. *epidermidis* PS10 biofilms during growth in chamber slides. Fig 2D and 2E demonstrate that not all biofilm-engaged bacteria undergo RsaE transcription although all bacteria in the population carry the plasmid (Fig 2F). Instead, RsaE is expressed in distinct spatial foci, and time lapse video recordings highlight the spatiotemporal *rsaE* promoter activity within the growing biofilm consortium (S1 Video). Staining with Ethidium Homodimer III (indicative for eDNA and/or dead cells) revealed localized bacterial lysis and eDNA release (Fig 2E; S2 Video). Furthermore, carbohydrate-specific fluorescence staining of the PIA biofilm matrix (using Alexa Fluor 488 wheat-germ agglutinin) visualized PIA production of the bacterial biofilm community (Fig 2E).

### RsaE favours PIA-mediated biofilm matrix expression and eDNA release

**Fig 3:**
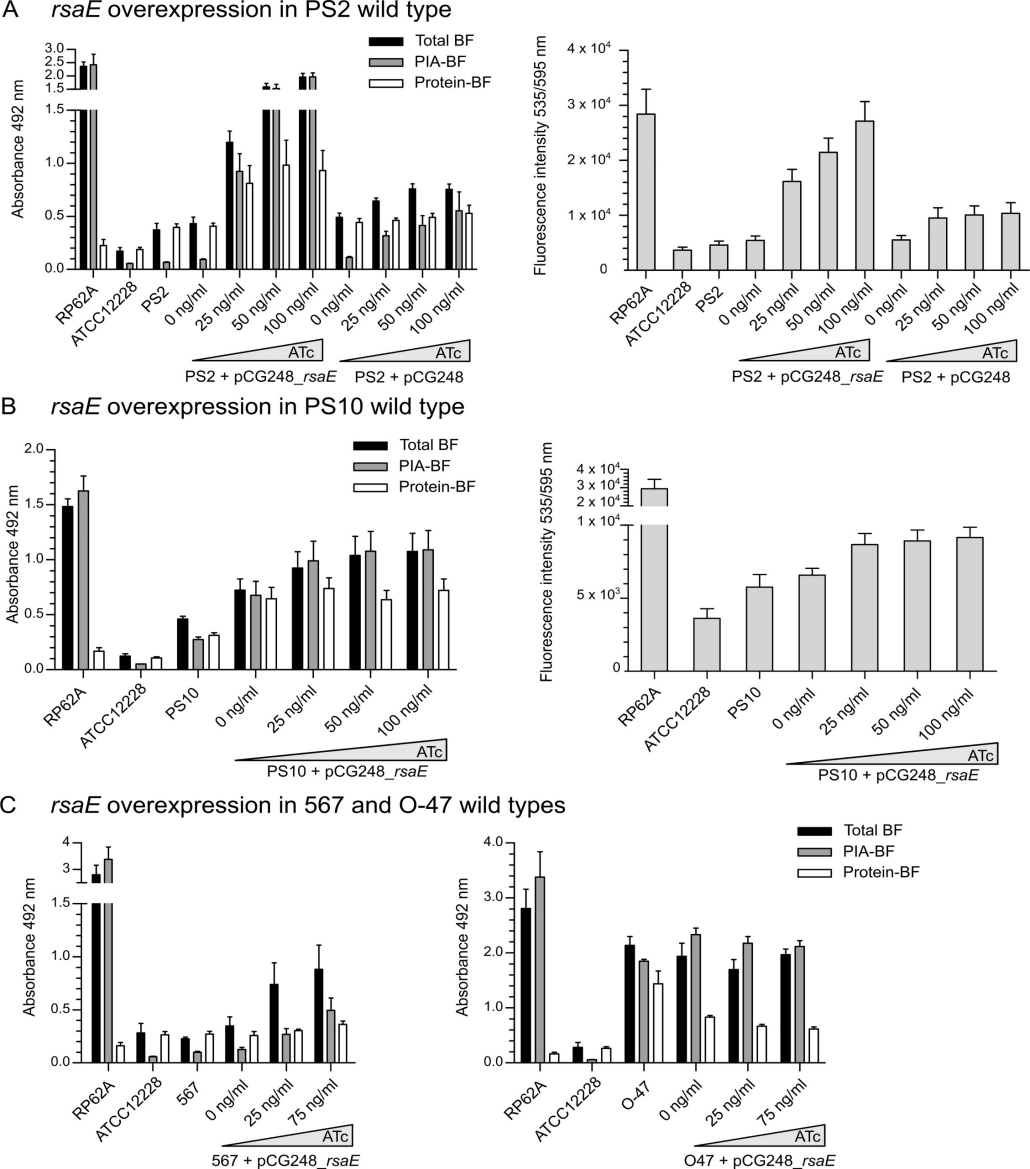
RsaE effects on biofilm production and eDNA release. (*A*) Left panel. Analysis of biofilm production of *S*. *epidermidis* PS2 (pCG248_*rsaE)* by static 96-well microtiter plate biofilm assays. Plasmid pCG248_*rsaE* harbours the *rsaE* gene under the control of an anhydrotetracycline (ATc)-inducible promoter. As control, a strain carrying an empty plasmid (pCG248) was included in the analyses. Expression of *rsaE* was induced by increasing ATc concentrations (25 to 100 ng/ml). Total biofilm (BF) mass as well as PIA- and protein-mediated biofilm proportions were determined by sodium-periodate and proteinase K treatments, respectively, as described in Methods. Sterile TSB medium served as background control. The strong PIA-producer *S*. *epidermidis* RP62A (*ica* locus positive) as well as *S*. *epidermidis* ATCC12228 (*ica* locus negative) were used as positive and negative controls, respectively. Biofilm production was also determined in the *S*. *epidermidis* PS2 wildtype. (*A*) Right panel. Detection of eDNA content in *S*. *epidermidis* biofilms by Ethidium Homodimer III staining and fluorescence intensity measurements at 535/595 nm. Biofilms were grown in 96-well microtiter plates using the same strains and conditions as in the left panel. (*B*) Analysis of biofilm production and eDNA release of *S*. *epidermidis* PS10 (pCG248_*rsaE)* by static 96-well microtiter plate biofilm assays. Biofilms were grown in 96-well microtiter plates using the same conditions as in panel A. *(C)* Analysis of biofilm production of *S*. *epidermidis* 567 and O47 carrying plasmid pCG248_*rsaE* by static 96-well microtiter plate biofilm assays. Biofilms were grown in 96-well microtiter plates using the same conditions as in panel A.

To study a possible influence of RsaE on eDNA release and PIA production in more detail, we cloned *rsaE* under the control of the anhydrotetracycline (ATc)-inducible promoter on vector pCG248 [24], which was transformed into the (PIA-negative) protein biofilm matrix producer PS2. In microtiter plate biofilm assays, ATc-mediated induction of RsaE expression resulted in an increase of the total biofilm mass in an ATc dose-dependent manner (Fig 3A, left). Differentiation of the biofilm matrix composition (by protease and sodium periodate treatments) revealed that the RsaE-induced biofilm in PS2 mainly consists of PIA, suggesting that RsaE supports PIA production and is able to trigger a PIA-biofilm phenotype upon overexpression in PS2 (Fig 3A, left). Further, RsaE overexpression triggered a dose-dependent increase of eDNA release in this strain (Fig 3A, right). We also transformed the RsaE overexpression plasmid into isolate PS10 and two other *ica-*positive *S*. *epidermidis* wildtype backgrounds (*i*.*e*. 567 and O-47). Upon RsaE overexpression, we recorded increased PIA-mediated biofilm production in strains PS10 and 567 (Fig 3B and 3C). In *S*. *epidermidis* O-47, however, PIA production did not change, suggesting strain-specificity of the RsaE effect on *S*. *epidermidis* biofilm formation (Fig 3C, right).

### RsaE gene deletion affects PIA-mediated biofilm production and eDNA release

**Fig 4:**
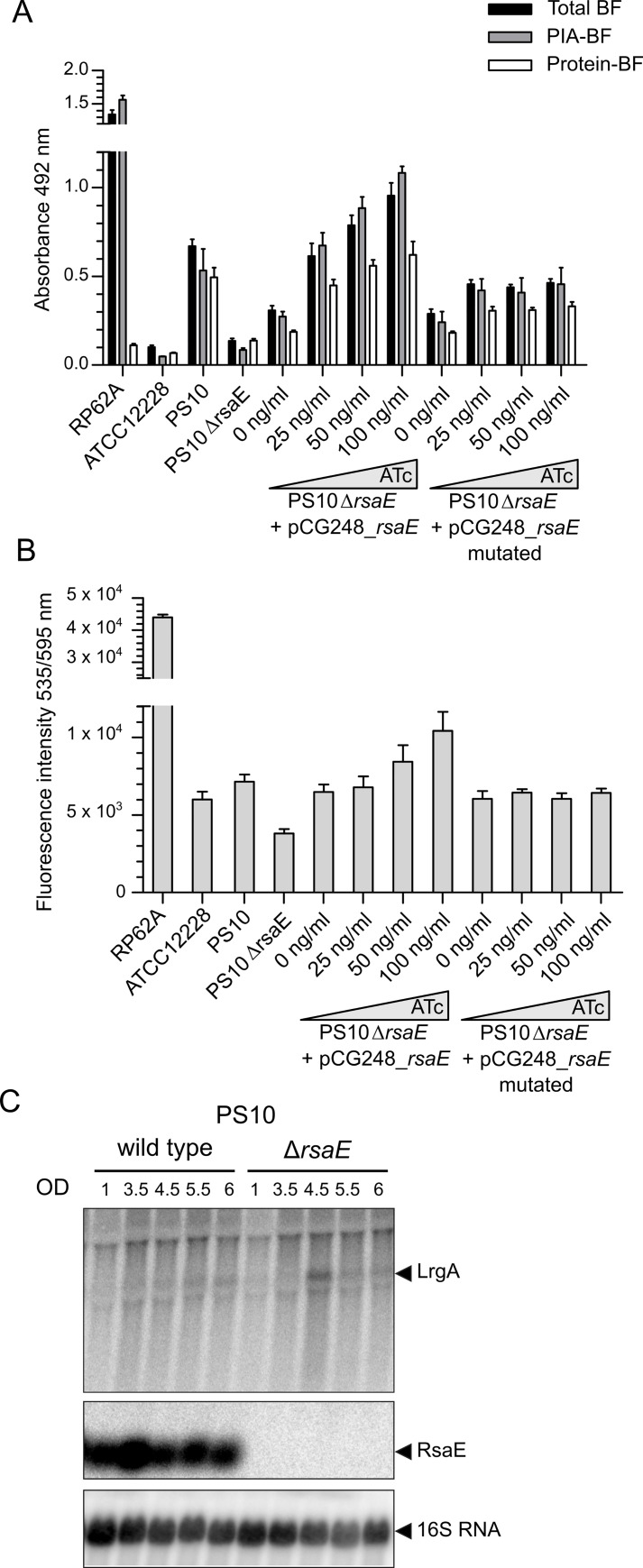
Effect of RsaE deletion on biofilm production and eDNA release. *(A)* Analysis of biofilm production of *S*. *epidermidis* PS10 wildtype, *rsaE* deletion mutant and complemented strains carrying either a wildtype copy of *rsaE* or a mutated version of RsaE (pCG248_*rsaE*_mutated) under ATc-inducible *tet* promoter control, respectively. Expression of *rsaE* was induced by increasing ATc concentrations (25 to 100 ng/ml). Total biofilm (BF) mass as well as PIA- and protein-mediated biofilm proportions were determined by sodium-periodate and proteinase K treatments, respectively, as described in Methods. Sterile TSB medium served as background control. The strong PIA-producer *S*. *epidermidis* RP62A (*ica* locus positive) as well as *S*. *epidermidis* ATCC12228 (*ica* locus negative) were used as positive and negative controls, respectively. *(B)* Detection of eDNA content in *S*. *epidermidis* biofilms by Ethidium Homodimer III staining and fluorescence intensity measurements at 535/595 nm. Biofilms were grown in 96-well microtiter plates using the same strains and conditions as in the panel A. *(C)* Northern blot detection of *rsaE* and *lrgA* expression during growth of *S*. *epidermidis* PS10 wildtype and *rsaE* deletion mutant in batch culture at 37°C in TSB. Optical densities (OD_600_) at which samples were taken are indicated.

To further test the putative influence of RsaE on PIA production, we generated a (markerless) *rsaE* gene deletion mutant in the PIA-producing *S*. *epidermidis* PS10 isolate. Compared to the wildtype, the *S*. *epidermidis* PS10 Δ*rsaE* mutant was strikingly impaired in PIA-mediated biofilm production and eDNA release (Fig 4A and 4B). Expression of RsaE on an ATc-inducible plasmid in the PS10 Δ*rsaE* mutant background was able to rescue PIA production and eDNA release in an ATc dose-dependent manner which even exceeded wild type PIA and eDNA levels (Fig 4A and 4B). To control for the specificity of the RsaE effect, a mutated version of RsaE was generated in which the C-rich motifs 1–3 (indicated in Fig 2B), which are supposed to mediate RsaE/ mRNA target interaction, were exchanged to A-stretches. No effect on PIA and eDNA was detectable in PS10 Δ*rsaE* when the mutated RsaE version was overexpressed from the plasmid (Fig 4A and 4B). To address any alleged strain-specific RsaE effect, we also generated an *rsaE* deletion mutant in the *S*. *epidermidis* O-47 background in which RsaE overexpression had shown no effect on biofilm formation (Fig 3C). In agreement with these findings, neither deletion of *rsaE* nor its overexpression in the mutant did influence PIA production and eDNA release in strain O-47 (S2 Fig). From the combined data we conclude that RsaE specifically influences PIA-mediated biofilm formation and eDNA release in *S*. *epidermidis*. Interestingly, this effect is strain-specific and does not occur in all *ica*-positive *S*. *epidermidis* backgrounds.

### The *cidA/lrgA* holin/antiholin system is expressed in *S*. *epidermidis* biofilms

**Fig 5:**
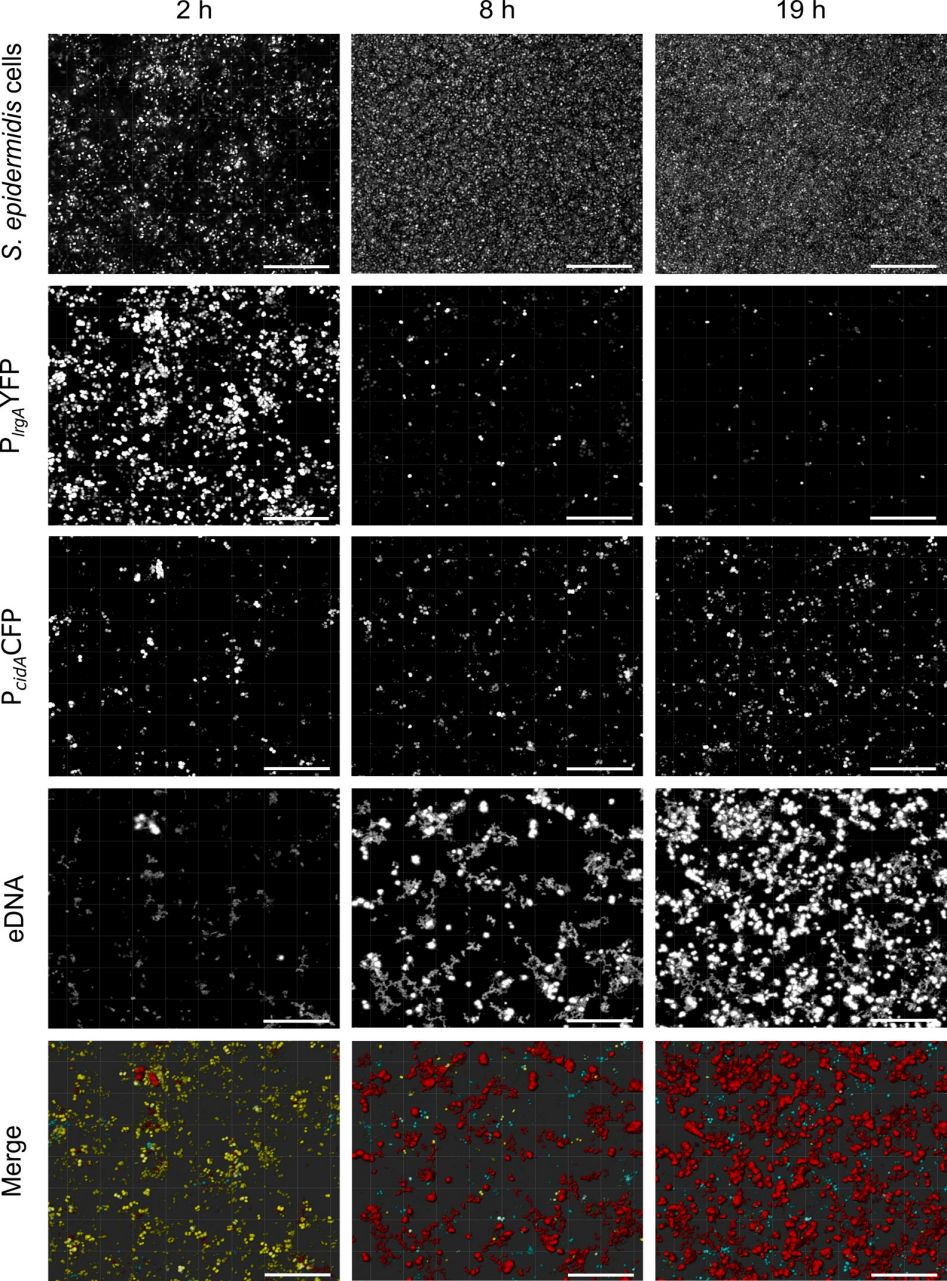
CLSM live cell imaging of *lrgA/cidA* expression and eDNA release in a *S*. *epidermidis* biofilm during growth in chamber slides. The strain harbours plasmid p_(P_*cidA*_CFP/P_lrgA_YFP) carrying the *lrgA* promoter fused to *yfp* (encoding yellow-fluorescent protein) and the *cidA* promoter fused to *cfp* (encoding blue-fluorescent Cerulean protein). eDNA was stained in red by Ethidium Homodimer III. Bars represent 20 μm.

The results obtained so far suggest that RsaE facilitates eDNA release, most probably by triggering lysis of *S*. *epidermidis* bacterial cells. In *S*. *aureus*, death and lysis were previously shown to play a functional role in biofilm development through the release of eDNA as biofilm matrix component [25, 26]. This effect is mediated by the products of the *S*. *aureus cid* and *lrg* operons in which *cidA* and *lrgA* encode holin and antiholin proteins, respectively [27]. Similar to phage holins, CidA and LrgA form membrane-associated complexes with CidA acting as lysis-triggering holin. LrgA is supposed to function as an antiholin protein that counteracts the lytic function of CidA [27]. In *S*. *epidermidis*, the *cidAB* and *lrgAB* operons are conserved, and in the dRNA-seq experiment the *lrgAB* genes appeared as differentially expressed between the PS2 and PS10 isolates (S1 Table). These observations prompted us to study *lrg* expression in *S*. *epidermidis* in more detail. We constructed a fluorescence reporter gene plasmid carrying the *lrgA* promoter fused to yellow-fluorescent protein gene *yfp* and the *cidA* promoter to *cfp* (cerulean). CLSM monitoring of YFP and CFP expression in combination with eDNA staining revealed *cidA* and *lrgA* promoter activity in a growing *S*. *epidermidis* biofilm, with only a part of the population expressing the genes (Fig 5). In the early growth stage (after two hours), promoter activity of the *lrgA* antiholin gene predominated with only a few bacteria expressing the *cidA* holin gene and undergoing lysis/ eDNA release (Fig 5, left panel). The situation changed with progressing biofilm development and maturation. After eight hours, and even more pronounced after 19 hours of growth, *cidA*-mediated holin expression increased, while *lrgA* expression was almost totally silenced. This effect was accompanied by an increased detection of eDNA within the maturing biofilm consortium (Fig 5, middle and right panels).

### RsaE binds to the *lrgA* antiholin gene mRNA

**Fig 6:**
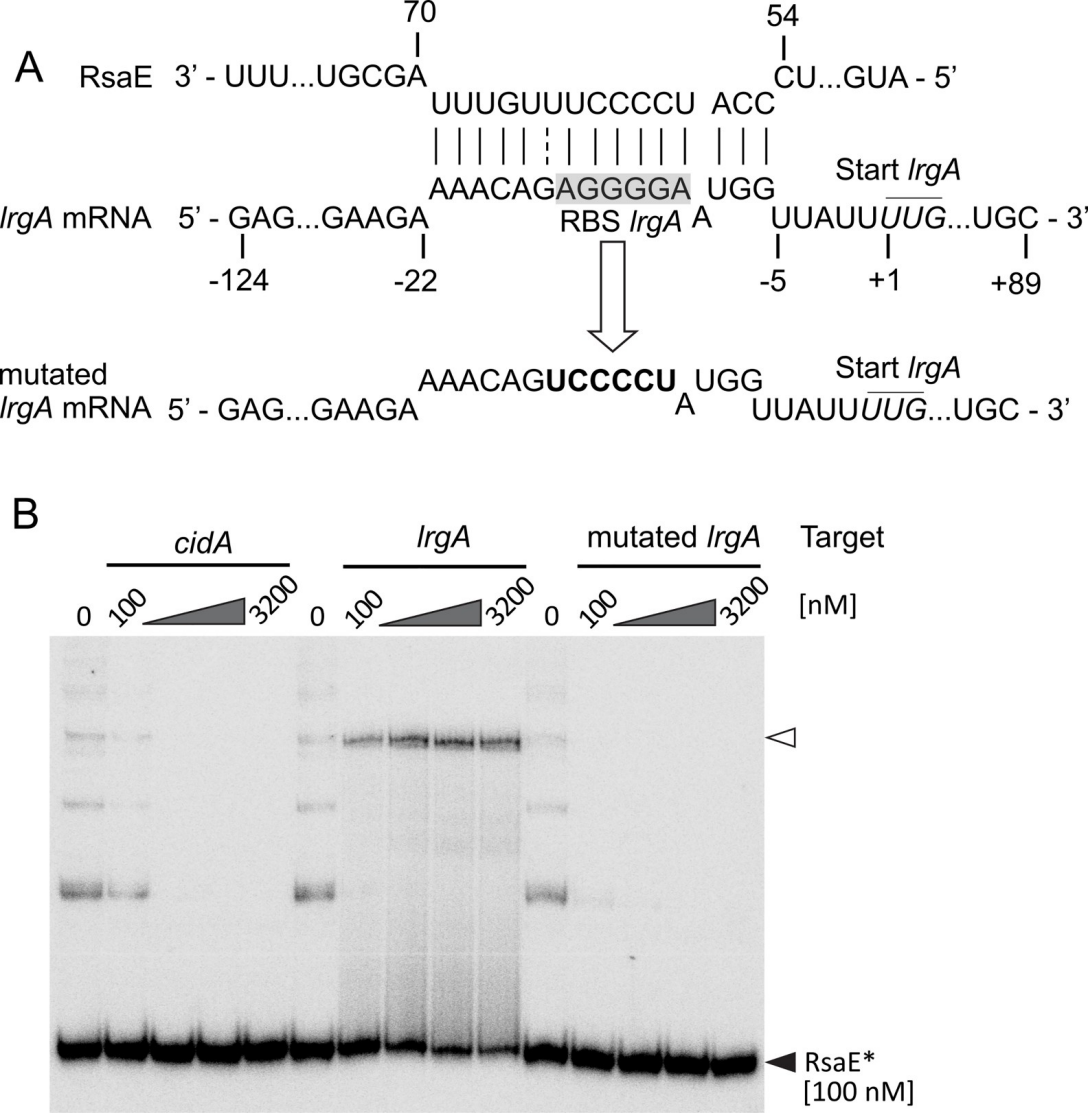
Interaction of RsaE with *lrgA* mRNA. (*A*) IntaRNA-based interaction prediction [29, 30] of *lrgA* mRNA with RsaE (top) as well as nature and positions of the nucleotide exchanges in the mutated *lrgA* mRNA (bottom). Numbering refers to the upstream (-124) and downstream (+89) region around the *lrgA* mRNA start codon (+1). Ribosomal binding site (RBS) of *lrgA* is highlighted in grey. (*B*) RNA/RNA electromobility gel shift assays (EMSAs) employing 100 nM of radioactively labeled (*) RsaE and increasing amounts of *cidA* (left), *lrgA* (middle) and mutated *lrgA* (right) target RNAs as binding partners. Open and filled triangles mark RsaE/target RNA complexes and labeled unbound RsaE, respectively.

Next, we addressed the question if RsaE might be capable of directly influencing *cidA/lrgA* expression in *S*. *epidermidis*. Usually, sRNAs exert their regulatory effects on gene expression by specific binding to target mRNAs. These interactions often occur in 5'-untranslated regions (5'-UTRs) of mRNAs and may involve ribosomal binding sites (RBS) and start codon regions, thereby negatively or positively influencing translation of the respective target genes [28]. In *S*. *aureus*, RsaE was shown to adopt a structure that exposes C-rich sequence motifs (red highlights in Fig 2B) which are prone to bind G-rich regions, including RBS in 5'-UTRs [19]. For RsaE, this interaction was shown to prevent the formation of ribosomal initiation complexes, resulting in negative effects of RsaE on target mRNA translation and stability [19, 20]. To get a first insight into putative RsaE/ mRNA target interactions in *S*. *epidermidis*, we employed the IntaRNA algorithm for *in silico* RsaE target prediction using *S*. *epidermidis* RP62A as reference genome (http://rna.informatik.uni-freiburg.de/IntaRNA; [29, 30]). Based on free energy and p-values, *lrgA* mRNA was identified as a high-score hit (ΔG-17.8 kcal/mol; p<0.003) (Fig 6A; S3 Table). To experimentally verify RsaE/mRNA target interactions, we performed electrophoretic mobility shift assays (EMSA) with *in-vitro*-transcribed RsaE and *lrgA* as well as *cidA* mRNAs as reaction partners (covering -124 to +89 nucleotides for *lrgA* and -143 to +62 nucleotides for *cidA* relative to their respective start codons). Incubation of a constant amount of radioactively labeled RsaE with increasing amounts of unlabeled *lrgA* mRNA resulted in RsaE binding which was reflected by a dose-dependent shift, while the same experimental setup using *cidA* mRNA as interaction partner had no effect (Fig 6B, middle and left panels). The IntaRNA approach predicted the RBS region of the *lrgA* mRNA to interact with a long unpaired nucleotide stretch (nt 55 to 69) of RsaE (Fig 6A). This region separates the two RsaE stem-loops and harbours two of the conserved C-rich motifs, from which one (motif 3, nt 59–62) is supposed to take part in *lrgA* mRNA binding (Fig 2B). To validate this interaction, we exchanged the respective nucleotides, covering the RBS of *lrgA*, from AGGGGA to UCCCCU (Fig 6A). No interaction with RsaE was detectable when we used the mutated *lrgA* mRNA as a binding partner, demonstrating that this motif is indeed critically involved in RsaE/*lrgA* mRNA interaction (Fig 6B, right panel). Further, addition of an antisense RNA oligonucleotide, covering the long unpaired RsaE nucleotide stretch, blocked RsaE/*lrgA* mRNA binding in a dose-dependent manner (S3B Fig). Finally, use of excess tRNAs as unspecific competitors did not prevent RsaE/*lrgA* mRNA complex formation, while addition of unlabeled RsaE diminished the signal strength in a dose-dependent manner, demonstrating specificity of the interaction (S3A Fig). Interestingly, when using *S*. *aureus lrgA* mRNA as RsaE target, no interaction was detectable between the partners (S3C Fig), and comparison of the nucleotide sequences revealed numerous nucleotide differences between the two species which also involved the RsaE binding site (S3D Fig). The combined data suggest that RsaE binds specifically to the 5'-UTR of the *S*. *epidermidis lrgA* mRNA involving the *lrgA* RBS and a conserved C-rich motif of RsaE.

### RsaE expression influences *lrgA* mRNA levels

The detected *in vitro* RsaE/ *lrgA* mRNA interaction, prompted us to test whether or not RsaE also influences *lrgA* mRNA levels in the staphylococcal cell. For this purpose, we hybridized the RNA samples from PS2 and PS10 also with *lrgA*-specific probes (Fig 2C, bottom). The Northern blot revealed that in PS10, no *lrgA* mRNA was detectable during the entire growth of the culture. Concomitantly, RsaE expression was constantly high in this strain (Fig 2C, upper panel). In contrast, except for early exponential and late stationary growth, *lrgA* mRNA was (weakly) detectable in the PS2 isolate, with a peak occurring during exponential growth at an OD_600_ of 5.4 (Fig 2C, bottom). Here, RsaE expression had dropped substantially in comparison to the previous RNA sampling point (i.e. OD_600_ of 4) at which only weak amounts of *lrgA* mRNA were detectable (Fig 2C), suggesting a negative correlation between RsaE and *lrgA* expression. However, as this might also be due to unrelated differences in RsaE and *lrgA* expression, we further tested the PS10Δ*rsaE* deletion mutant for putative effects on *lrgA* expression. In this Northern blot experiment, the PS10 wildtype did not exhibit *lrgA* mRNA-specific signals (Fig 4C). In the PS10Δ*rsaE* mutant strain, however, *lrgA* mRNA was detectable in the exponential growth stage at an OD_600_ of 4.5 Fig 4C), which resembles the *lrgA* expression pattern of the PS2 isolate (Fig 2C). From this experiment we conclude that RsaE indeed influences *lrgA* mRNA levels and that a negative correlation exists between (high) RsaE expression and (low) *lrgA* mRNA levels in the *S*. *epidermidis* PS2 and PS10 strains.

### RsaE undergoes processing at the 5'-end and exists as an additional truncated species

**Fig 7:**
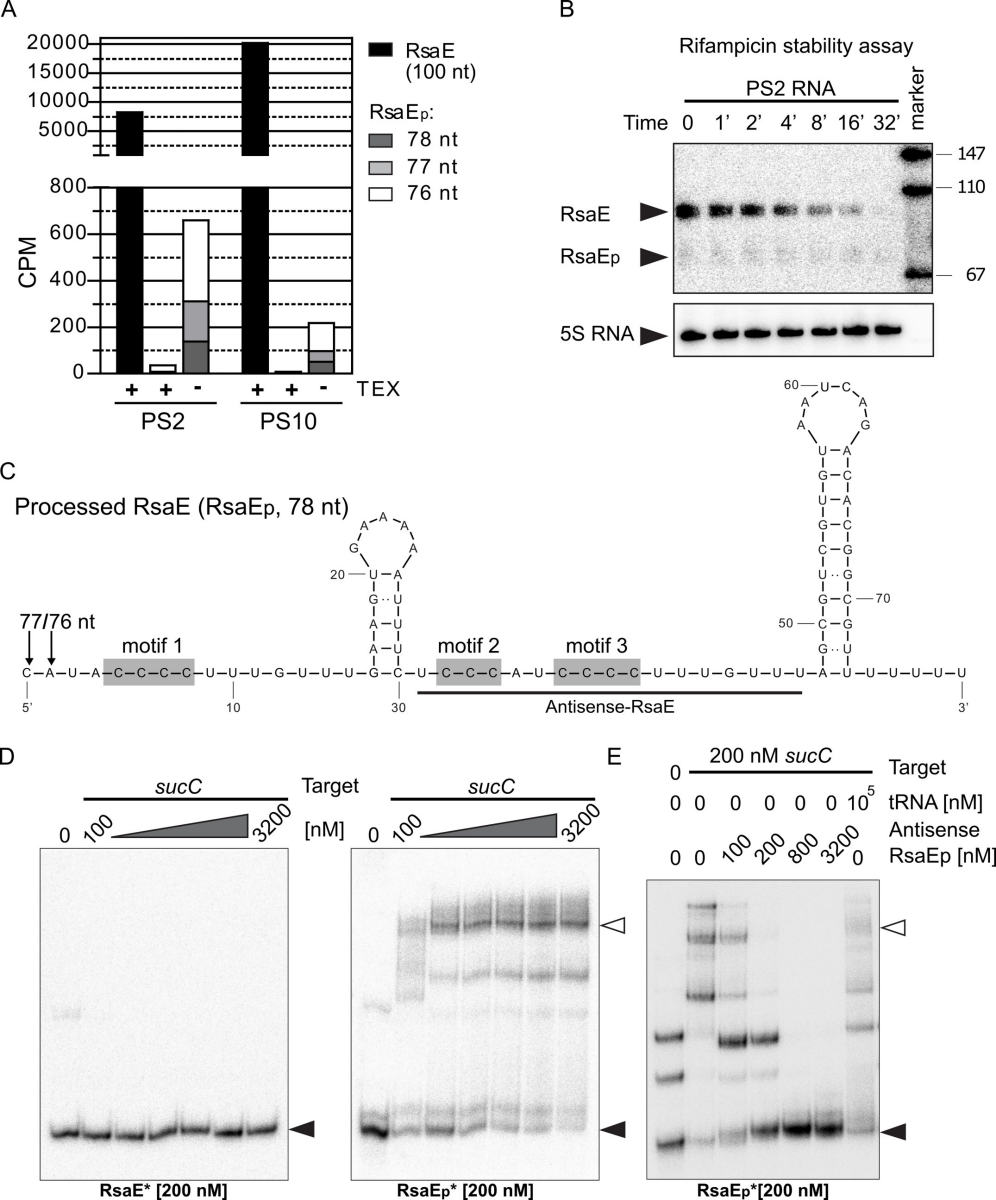
RsaE processing, stability and interaction with *sucC* mRNA. (*A*) Quantification of full-length and processed RsaE (RsaE_p_) transcripts in PS2 and PS10 by dRNA-seq analysis, with (+) and without (-) TEX treatment. CPM: counts per million reads, calculated as NOAR (number of aligned reads)*10^6^/TNOAR (total number of aligned reads). Black columns indicate the number of transcription starts of full-length RsaE (100 nt); the number of processed RsaE species (RsaE_p_, 76–78 nt) are shown as white, light grey and dark grey columns, respectively. (*B*) Stability determination of full-length (RsaE) and processed (RsaE_p_) RsaE species. *S*. *epidermidis* PS2 was grown in TSB to early-exponential growth stage and RNA was isolated before (0) and at the time points indicated after transcription blocking by rifampicin. RNA samples were subjected to Northern blot analyses using radioactively labeled ssDNA-oligonucleotide probes targeting either RsaE or 5S rRNA as loading control. (*C*) Proposed secondary structure of the processed RsaE species (RsaE_p_, 78 nt) according to mFold-4.7-based prediction [65]. Grey boxes highlight positions of the C-rich motifs within the processed RsaE molecule. Nucleotides complementary to the antisense-RsaE RNA probe used in (D) are underlined. Arrows indicate alternative RsaE processing sites identified by dRNA-seq. (*D*) EMSAs using increasing amounts of *sucC* target RNA and radioactively labeled (*) full-length (RsaE, left panel) or processed RsaE (RsaE_p_, right panel) as binding partners. (*E*) *sucC*/RsaE_p_ EMSA upon competition with an antisense-RsaE RNA oligonucleotide or unspecific tRNAs during complex formation. 200 nM of *sucC* target RNA was mixed with increasing amounts of antisense-RsaE RNA oligonucleotide (lanes 3–6) or a 500-fold excess of yeast tRNAs (lane 7) prior to addition of 200 nM radioactively (*) labeled RsaE_p_ to the samples. Filled triangles in (D) and (E) indicate labeled unbound RsaE and open triangles mark RsaE/target complexes.

When analyzing Northern blots with RNA obtained from *S*. *epidermidis* PS2 and PS10, we regularly noticed appearance of a faint additional RsaE-specific band which was approximately 20 nucleotides smaller than the 100-nucleotide main transcript (Fig 2C*)*. Detailed inspection of the dRNA-seq data set confirmed existence of additional smaller RsaE species which lacked 22 to 24 nucleotides at the 5’- end of the sRNA in both the PS2 and PS10 variants (Fig 7A). In terminator-5'-phosphate-dependent exonuclease (TEX)-treated RNA samples, however, the truncated RsaE versions were no longer detectable (Fig 7A). TEX degrades RNA molecules with a 5'-monophosphate, which usually occur upon RNA processing, but not RNA molecules carrying a 5'-triphosphate which are typically generated upon primary transcription [31]. Hence, the data suggest that the shorter RNA species are likely to be derived from full-length RsaE by RNA processing rather than by transcription re-initiation at *rsaE* positions 22 to 24 (Fig 7A). Next, a rifampicin assay was performed to test for RsaE stability. For this purpose, *de novo* transcription was blocked by adding the RNA polymerase inhibitor rifampicin to a growing *S*. *epidermidis* PS2 culture that had reached the early exponential growth stage. Total RNA was then isolated at different time points after rifampicin addition and subjected to Northern blot analysis. Fig 7B indicates that the two RsaE species are still detectable 16 to 32 minutes after transcription arrest, suggesting longevity and coexistence of both the full-length and the processed sRNA molecules (Fig 7B).

### Processed RsaE recognizes different mRNA targets than full-length RsaE

Interestingly in *Bacillus subtilis*, where RsaE is also conserved, the sRNA was recently shown to undergo RNase Y-driven processing at the 5'-end [32]. Removal of 20 nucleotides resulted in expansion of the RsaE mRNA target repertoire in this organism, including the succinyl-CoA synthetase-encoding *sucCD* mRNA employed in the TCA cycle [32]. In *S*. *aureus*, *sucC* mRNA was also identified as an RsaE target resulting in inhibition of initiation complex formation [18]. When testing *S*. *epidermidis sucC* mRNA for binding to full-length RsaE in our experiments, no interaction between the two RNAs was detectable by EMSAs (Fig 7D, left panel). However, using the processed RsaE species (RsaE_p_) in the same approach revealed binding and formation of multiple *sucC* mRNA/RsaE_p_ complexes (Fig 7D). This interaction proved to be specific as addition of an RsaE-specific anti-sense oligonucleotide to the *sucC* target inhibited *sucC*/ RsaE_p_ complex formation in a dose-dependent manner (Fig 7E), while competition with an excess of unspecific tRNA was unable to prevent complex formation (Fig 7E). Also, *sucD* mRNA (the second cistron of the *sucCD* operon) was found to interact with processed RsaE_p_, but not with full-length RsaE (S4 Fig), suggesting that RsaE processing results in recognition of additional mRNA targets which also comprise TCA cycle-engaged mRNAs.

### Processed RsaE interacts with *icaR* mRNA encoding the repressor of PIA biofilm production

**Fig 8:**
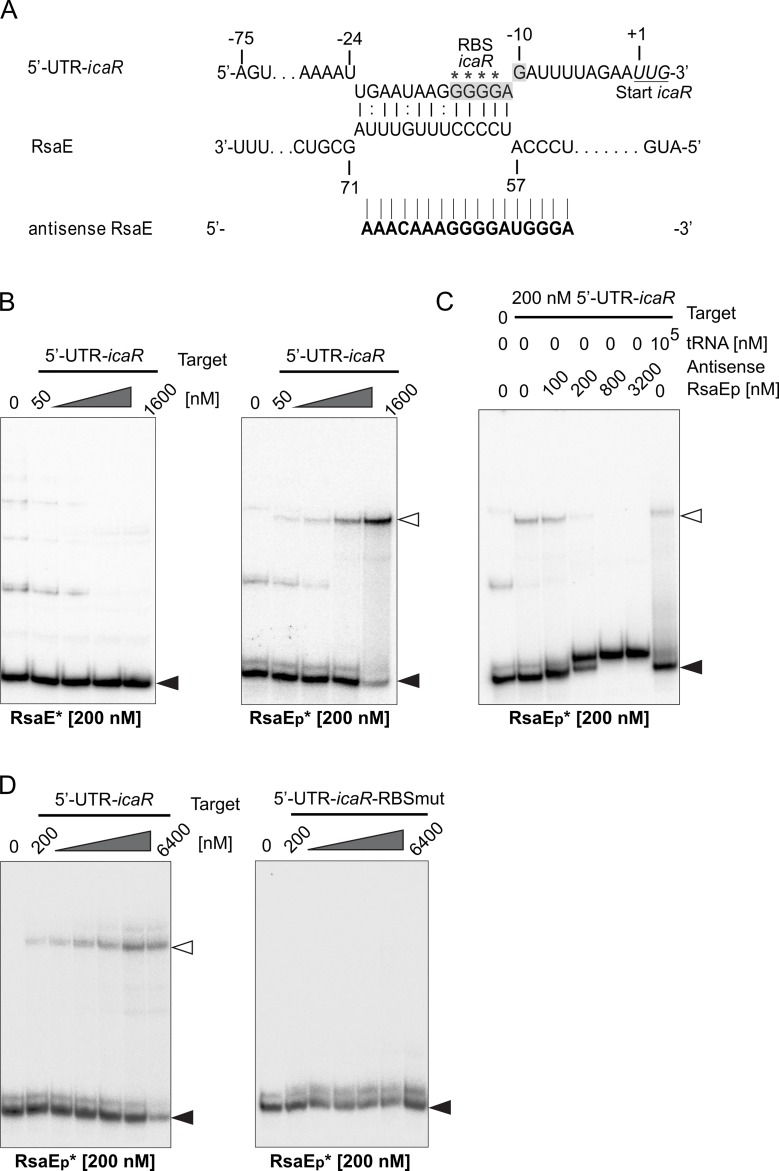
Interaction of processed RsaE (RsaE_p_) with 5'-UTR of *icaR*. (*A*) IntaRNA-based prediction of interaction between RsaE and the 5'-UTR-*icaR* [29, 30]. The *icaR* ribosomal binding site (RBS) is highlighted in grey and the *icaR* start codon is underlined. Asterisks mark G to U mutations used to create 5'-UTR-*icaR*-RBS-mut in (D). The nucleotide sequence of the antisense RsaE RNA oligonucleotide is shown at the bottom. (*B*) EMSAs using increasing amounts of 5'-UTR-*icaR* target RNA and radioactively labeled (*) full-length (RsaE, left panel) or processed RsaE (RsaE_p_, right panel) as binding partners. (*C*) EMSA with 5'-UTR-*icaR* and RsaE_p_ upon competition with an antisense-RsaE RNA oligonucleotide or unspecific tRNAs during complex formation. 200 nM of the 5'-UTR-*icaR* target RNA was mixed with increasing amounts of antisense-RsaE RNA oligonucleotide (lanes 3–6) or a 500-fold excess of yeast tRNAs (lane 7) prior to addition of 200 nM radioactively (*) labeled RsaE_p_ to the samples. (*D*) EMSA employing 200 nM radioactively (*) labeled RsaE_p_ with increasing amounts of either wildtype 5'-UTR-*icaR* (left) or 5'-UTR-*icaR*-RBS-mut (right) carrying mutations in the ribosomal binding site of *icaR* mRNA (GGGG to UUUU, marked by asterisks in (A)). Filled triangles in B-D indicate labeled unbound RsaE and open triangles mark RsaE/target complexes.

IntaRNA-based mRNA target prediction identified, amongst numerous other genes, also the region upstream of the *icaR* gene as a putative RsaE interaction partner (Fig 8A, S3 Table). *icaR* encodes a TetR-family transcription factor that binds to the *icaADBC* promoter region and acts as immediate repressor of PIA-mediated biofilm production [33]. Analysis of the dRNA-seq data of *S*. *epidermidis* PS2 and PS10 revealed presence of an *icaR* 5'-UTR whose transcription start mapped 74 base pairs upstream of the *icaR* TTG start codon, which is in good agreement with the previously identified size of the *icaR* 5'-UTR in *S*. *aureus* which is 72 nt in length [34]. The predicted interaction region of the *S*. *epidermidis icaR* 5'-UTR with RsaE involved the ribosomal binding site of *icaR* (Fig 8A), but when using full-length RsaE as a binding partner, no interaction was detectable in EMSA experiments (Fig 8B, left panel). In contrast, employing processed RsaE in the assay resulted in interaction between the shorter RsaE_p_ species and the *icaR* 5'-UTR (Fig 8B, right panel). The complexes even formed in the presence of a 500-fold excess of unspecific tRNAs as competitors, while their formation was inhibited in a dose-dependent manner upon competition with an RsaE-specific antisense RNA oligonucleotide (Fig 8C), suggesting specificity of the interaction between processed RsaE and *icaR* 5'-UTR. Introducing mutations into the RBS of *icaR* 5'-UTR (GGGG to UUUU), resulted in complete loss of complex formation with processed RsaE, demonstrating that processed RsaE binding critically involves the RBS of *icaR* mRNA (Fig 8D). These findings led us to conclude that processed RsaE has the capacity to directly influence PIA-mediated biofilm-formation by interaction with its repressor *icaR*.

## Discussion

### Gene expression in biofilm matrix variants is heterogeneous

The *S*. *epidermidis* PS strain used in this study is characterized by its pronounced phenotypic variability with respect to biofilm matrix production [17]. Heterogeneous bacterial populations are generally thought to have a fitness advantage in comparison to homogenous populations [35]. Heterogeneity may be generated by genetic mechanisms which impose an 'insurance effect' to bacterial populations through the generation of well adapted genetic variants fit to withstand a plethora of adverse conditions [36, 37]. In the course of an infection, this may result in the selection of specialized variants capable to resist host defense mechanisms or antibiotics. Diversification into cell types with varying phenotypes, however, can also be achieved by regulatory circuits as well as by stochastic and bistable variations in gene expression patterns [35]. This phenotypic heterogeneity seems to be particularly important for biofilm-forming bacterial communities in which the process not only provides a hedge against unpredictable changes in the environment, but also drives division of labor between matrix-producing and non-producing variants [38, 39]. Thus, phenotypic heterogeneity seems to represent a general inbuilt feature of biofilm communities which supports the formation of the biofilm architecture itself by meeting the varying metabolic challenges that exist within different microniches of the biofilm [38, 40, 41]. Indeed, the differences in gene expression observed between PS2 and PS10 by the dRNA-seq approach reflect very well the metabolic status of a non-PIA versus a PIA-biofilm matrix producer. Thus in PS10, downregulation of TCA cycle and other central carbon metabolism genes would allow for the redirection of carbon sources into aminosugar and PIA precursor synthesis as previously demonstrated for *S*. *epidermidis* (Fig 1) [21]. Also, decreased transcription of the *agr* quorum-sensing system and phenol soluble modulin genes are in good agreement with the known roles of these factors in PIA matrix biofilms (Fig 1; S1 Table) [42, 43]. The most surprising and novel features revealed by the dRNA-seq approach, however, were the affection of the *lrg/cid* system (see below) and the differential expression of RsaE, with PS10 displaying higher RsaE expression than PS2 (Fig 2C; Fig 7A). The observed variations in carbon utilization patterns together with a previously described role of RsaE in the control of central carbon metabolism in *S*. *aureus* prompted us to study RsaE functions in *S*. *epidermidis* in more detail [18, 19].

### RsaE is differentially expressed within biofilm communities

While the initial dRNA-seq experiment provided a transcription 'snapshot' of bulk RNA isolated in the exponential growth stage, the Northern blot and CLSM experiments revealed interesting additional information on RsaE transcription dynamics over time and within the spatial organization of the biofilm community. In batch cultures, RsaE expression in PS2 undergoes a growth stage-dependent control, while RsaE seems to be constitutively transcribed in PS10 (Fig 2C). Such variations in RsaE expression are apparently not uncommon. Thus, when testing other *ica*-positive *S*. *epidermidis* strains (*i*.*e*. 567 and O-47) for RsaE expression, we also found differences in RsaE transcription patterns suggesting high flexibility regarding expression of this sRNA in the species (S6 Fig). Interestingly, CLSM monitoring of RsaE expression within an *in situ*-grown PS10 biofilm community revealed that not all bacteria undergo RsaE expression. Instead, we found spatiotemporal activity of the *rsaE* promoter in distinct foci (Fig 2D, E; S1 and S2 Videos). A similar behaviour was recorded in planktonic cultures where biofilm-forming staphylococci tend to adhere to each other and form aggregates [44]. Here, RsaE expression was also heterogeneous and increased when bacterial cells started to aggregate in cell clusters (S7 Fig). The reasons for the varying RsaE expression both between strains as well as during biofilm organization remain elusive. Limited information is currently available on regulation of RsaE expression in *S*. *epidermidis*. However, RsaE is highly conserved among Firmicutes, and in *S*. *aureus* the Agr quorum-sensing system was suggested to be involved in RsaE control [19]. Recent data further demonstrate involvement of the oxygen/ nitric oxide-sensing two-component SrrAB system in RsaE regulation (both in *S*. *aureus* and in *Bacillus subtilis)* [32]. As SrrAB is known to repress Agr under low oxygen conditions (at least in *S*. *aureus*) [45, 46], it is tempting to speculate that the two regulators might act together to control RsaE in response to oxygen availability. In *B*. *subtilis*, RsaE (synonym RoxS) is activated by SrrAB (ResDE) and repressed by Rex which is a repressor controlling fermentation-associated genes by sensing NAD+/NADH levels [23]. Interestingly, ResD and Rex binding sites were found to be conserved within the promoter regions of various bacilli and staphylococci, including *S*. *aureus* and *S*. *epidermidis*, suggesting similar regulation and responsiveness to metabolic signals [22, 23]. Thus, varying carbon utilization patterns displayed by PIA-producing and non-producing micropopulations may trigger varying redox potentials within distinct biofilm microniches. It is conceivable that SrrAB/ Rex are sensitive to these conditions and subsequently mediate varying RsaE expression patterns. However, as RsaE itself is a proven regulator of central carbon metabolism, it is extremely difficult to distinguish here between cause and effect. RsaE influences numerous other metabolic functions as well, including a recently revealed repression of arginine degradation in *S*. *aureus* and facilitation of malate transport in *B*. *subtilis* [20, 23]. Thus, in an alternative scenario in which RsaE might undergo stochastic expression within *S*. *epidermidis* biofilm communities, this versatile switch may trigger local metabolic diversification, resulting in niches with varying carbon utilization and other metabolic patterns.

### RsaE facilitates PIA biofilm expression and eDNA release in a strain-specific manner

The data obtained so far support the idea of RsaE as a PIA-biofilm promotive factor. Thus, overexpression of RsaE in the otherwise PIA-negative PS2 variant prompted a PIA-biofilm phenotype (Fig 3A). The same effect was observed with *S*. *epidermidis* 567 which is another *ica-*positive wildtype strain displaying low PIA production (Fig 3C; S6 Fig). Also, RsaE was able to further increase PIA-mediated biofilm formation in the PIA-producing PS10 background (Fig 3B). Most strikingly, deletion of the *rsaE* gene in *S*. *epidermidis* PS10 resulted in loss of PIA production and diminished eDNA release in the mutant (Fig 4A and 4B). Moreover, complementation of the wildtype phenotype by intact RsaE, but not by a functionally inactive form of the sRNA suggests specific involvement of RsaE in PIA production and eDNA release in *S*. *epidermidis* PS10 (Fig 4A and 4B). Surprisingly, neither RsaE overexpression nor *rsaE* gene deletion had an effect on biofilm formation of strain *S*. *epidermidis* O-47 (Fig 3C; S2 Fig). The molecular background of the aberrant behaviour of RsaE in this isolate is currently not known. According to multi locus sequence typing (MLST), *S*. *epidermidis* O-47 belongs to the same MLST sequence type (*i*.*e*. ST2) as the other strains tested in this study. Also, the nucleotide sequences of *rsaE*, its putative targets and regulators (*i*.*e*. SrrAB, Rex) do not differ. However, due to a frameshift mutation in the *agrC* gene, strain O-47 represents a functional *agr* mutant [47]. Whether or not this lack of the quorum-sensing function causes the 'non-effect' of RsaE on biofilm formation in strain O-47 is an interesting question to be answered in the future. Together, the data suggest that RsaE represents a trigger of *S*. *epidermidis* biofilm matrix switching towards PIA production. The strain-specific RsaE effect revealed in this study highlights (once again) the huge flexibility that *S*. *epidermidis* displays with respect to gene regulation.

### RsaE interacts with the *cidA/lrgA* holin/antiholin system of *S*. *epidermidis*

The release of eDNA upon RsaE overexpression is a remarkable feature observed in this study (Fig 3A). eDNA has been recognized as a regular component and stabilizing factor in many bacterial biofilms, including *S*. *epidermidis* and *S*. *aureus*, and its release is considered to be mainly due to bacterial lysis [9, 48, 49]. In *S*. *aureus*, lysis is mediated and controlled by the CidA holin and LrgA antiholin proteins which were shown to be differentially expressed within *S*. *aureus* biofilms [26, 27, 40]. Also, the system is known to be involved in the generation of metabolically distinct microenvironmental niches during maturation and (programmed) structuring of *S*. *aureus* biofilms [50]. Expression of the *cidABC/ lrgAB* operons in *S*. *aureus* is strictly controlled by their cognate regulators (*i*.*e*. CidR and LytSR) which are sensitive to metabolic signals, with oxygen and carbon flow acting as potent triggers [51, 52]. CLSM monitoring of *cidA*/*lrgA* expression revealed that the genes undergo, similar to *S*. *aureus*, heterogeneous and dynamic expression in *S*. *epidermidis* as well (Fig 5). Further, *lrgA* was among the genes differentially expressed between PS2 and PS10, and the 5'-UTR of *lrgA* was experimentally verified to represent an RsaE binding partner (S1 Table; Fig 2C; Fig 6). This interaction critically involves the *lrgA* ribosomal binding site, suggesting a negative effect of RsaE on *lrgA* mRNA translation (Fig 6). Further, we found a negative correlation between RsaE expression and detection of *lrgA* mRNA in the *S*. *epidermidis* PS2 and PS10 strains (Figs 2C and 4C). As *lrgA* encodes an antiholin protein that counteracts the lytic activity of the CidA holin, it is reasonable to suggest that the increased eDNA release recorded upon RsaE overexpression is due to enhanced bacterial lysis as a result of the lacking LrgA-mediated antiholin activity, with the released eDNA further stabilizing the biofilm (S2 Video). Based on findings in *S*. *aureus* and *B*. *subtilis*, however, it is also conceivable that LrgAB might additionally facilitate biofilm formation via modulation of central carbon metabolism. Thus, in *S*. *aureus* programmed bacterial cell death through the Cid/ Lrg system was shown to be tightly linked to bacterial overflow metabolism with intracellular acidification and respiratory dysfunction as effector mechanisms [52]. In this CidR-controlled cascade, pyruvate is the crucial compound whose conversion into either acetate or acetoin influences bacterial cell death and biofilm mass [52]. Interestingly in *B*. *subtilis*, the LrgAB homologs PftAB were recently demonstrated to exhibit a (second) function as a pyruvate transport system whose expression is controlled by varying extra- and intracellular pyruvate levels [53]. If LrgAB would exert a similar function in pyruvate transport in *S*. *epidermidis*, the RsaE/ *lrgA* mRNA interaction might also influence intracellular pyruvate concentrations and affect carbon flux in favor of PIA synthesis (see below). However, at the present stage of experimental work this is mere speculation, the more so as major differences in the RsaE function seem to exist between bacterial species. Thus, we found the RsaE/*lrgA* mRNA interaction to be specific for *S*. *epidermidis*, but not to occur in *S*. *aureus* which was due to significant differences in the *lrgA* nucleotide sequence between the two species, including variations in the ribosomal binding sites involved in RsaE binding (S3 Fig). In agreement with our data, a recent study identified an AAGGGG motif (akin to RBS) as the predominant motif for RsaE interactions [20]. In fact, a very similar motif (AGGGGA) builts the RBS of *S*. *epidermidis lrgA* mRNA which differs from the canonical staphylococcal RBS present in *S*. *aureus lrgA* mRNA (AGGAGG) (S3 Fig). Fig 2B illustrates that RsaE is identical in *S*. *aureus* and *S*. *epidermidis* (the two nucleotide exchanges within the primary sequences do not alter the RsaE structure or take part in target binding) (Fig 2B). Therefore, non-binding of RsaE to *S*. *aureus lrgA* mRNA is likely to be due to a mismatch between the C-rich motifs of RsaE and the RBS of *lrgA* mRNA of *S*. *aureus* (S3 Fig). This finding certainly warrants further investigations as it suggests fundamental differences in the control of CidA/LrgA-mediated lysis between *S*. *epidermidis* and *S*. *aureus*.

### RsaE is processed resulting in extension of the mRNA target spectrum

In the course of our studies we found that RsaE undergoes processing at the 5'-end, generating a species shortened by approximately 20 nucleotides which coexists with full-length RsaE (Fig 2C; Fig 7A and 7B). This is very similar to *B*. *subtilis* in which RsaE was found to be processed by RNase Y (and degraded by RNase J1), revealing a processed species of the same length and sequence as in *S*. *epidermidis* [32]. Processing of sRNAs is not unusual and was described before in a number of sRNAs of Gram-negative bacteria such as SdsR, ArcZ and RprA in *E*. *coli* and Salmonella, [54–56]. Often, processed sRNA species represent the more abundant and functionally active sRNA form. In *S*. *epidermidis*, the processed form of RsaE appears as a minor species, but with comparable longevity as full-length RsaE. This suggests a certain potential for functionality of the processed form, rather than representing a transient degradation intermediate. Indeed, processing resulted in interaction of RsaE_p_ with *sucCD* and *icaR* mRNAs, functions that were not detectable with full-length RsaE (Fig 7C–7E, S4 and S5 Figs). This extension of the RsaE target spectrum is again reminiscent of *B*. *subtilis* in which processed RsaE was demonstrated to bind other targets than the full-length form, including a proven interaction with *sucCD* mRNA [32]. Deletion of the first 20 to 24 nucleotides of RsaE (arrows in Fig 2B) is likely to prompt the processed RsaE to adopt a different structure than the full-length sRNA, by abolishing the long stem 1 and moving the C-rich motif 1 (exposed in loop 1 of full-length RsaE) to a long unpaired stretch in the 5'-region of the sRNA (Fig 7C, S5 Fig). In concert with the two other C-rich motifs, this would offer more options for the interaction of the processed RsaE with G-rich regions in mRNA targets. With respect to *sucC* mRNA, opening of the RsaE structure would enable motif 1 to take part in additional base pairing interactions involving the early coding region of *sucC* (S5 Fig), which is in agreement with the situation described for *B*. *subtilis* RsaE/ *sucC* binding [32]. Similar might apply to processed RsaE binding to the 5'-UTR of *icaR* mRNA, although the structural details of this interaction are not that obvious. Based on bioinformatic predictions, both RsaE species would be prone to interact with *icaR* RBS through the region surrounding motif 3 of RsaE (Fig 8A). Experimentally, however, only processed RsaE was shown to bind to 5'-UTR *icaR* mRNA, and this interaction involved the RBS of *icaR* (Fig 8B–8D). The reasons why full-length RsaE does not recognize the RBS is not clear yet, and it is conceivable that additional structural constraints of the 5'-UTR of *icaR* mRNA may support processed RsaE binding. Further experimental work is currently under way to address this interesting question.

### Putative RsaE effects on PIA biofilm matrix production

RsaE has been shown to exert negative effects on mRNA targets, which is mainly due to the capacity of the C-rich motifs to mediate interaction with ribosomal binding sites of mRNAs, resulting in translation inhibition and decreased mRNA stability [18, 19, 32]. Given this negative role, binding of RsaE to *sucCD* and *icaR* mRNAs may explain the promotive effect of the sRNA on PIA biofilm matrix production. In case of *sucCD*, this is likely to occur indirectly via influencing the central carbon flow through the TCA cycle. PIA is a homopolymer consisting of beta-1,6 linked *N*-acetylglucosamine residues, whose precursor is UDP-*N*-acetylglucosamine (UDP-GlcNac). PIA production (and export) is a metabolically costly process requiring sufficient glucose or other C6 carbon sources to be fuelled into UDP-GlcNac synthesis. For *S*. *epidermidis* it was previously shown that repression of the TCA cycle is critical to enable PIA synthesis [21, 57]. Thus, PIA production was shown to occur exclusively during exponential growth when carbon flow through the TCA cycle is low. Blockage of the pathway (either by chemical inhibition or mutagenesis of corresponding genes) resulted in the redirection of carbon from energy gain and growth into UDP-GlcNac and eventually PIA synthesis [21, 57]. As TCA cycle stress was identified as a strong and potent signal in PIA synthesis control, it is reasonable to suggest that the detected interaction of processed RsaE with *sucCD* mRNA (and possibly also with other mRNAs of the pathway) represses TCA cycle activity and, as a result, triggers PIA synthesis. This effect may be further supported by binding of processed RsaE to the ribosomal binding site of *icaR* mRNA thereby blocking translation of the *icaADBC* operon repressor IcaR (Fig 8). Interestingly in *S*. *aureus*, *icaR* mRNA translation was recently shown to undergo autoregulation by an internal basepairing mechanism which does not occur with *S*. *epidermidis icaR* mRNA [34]. We currently speculate that RsaE might functionally replace the lacking autoregulation mechanism in *S*. *epidermidis*, but clearly more experimental work is required to substantiate this hypothesis. Together, these findings highlight remarkable differences regarding biofilm control between *S*. *epidermidis* and *S*. *aureus*.

### Conclusions

The combined data of this report suggest that in *S*. *epidermidis* biofilms RsaE facilitates production of two important biofilm matrix components, *i*.*e*. PIA and eDNA. RsaE mediates these effects by influencing central carbon metabolism, bacterial lysis as well as biofilm gene expression control. These mechanisms make RsaE a likely candidate to trigger metabolic diversity and division of labor in *S*. *epidermidis* biofilm communities, the more so as RsaE itself is heterogeneously expressed within biofilm populations. In this respect it is conceivable that RsaE-mediated localized bacterial lysis might play a particular role, not only by releasing eDNA as a stabilizing biofilm matrix component, but also by providing nutrients to non-lysing and surviving cells in the immediate neighborhood. Thus, RsaE expression (and subsequent CidA-mediated lysis) could be regarded as a form of bacterial altruism contributing to biofilm structuring and survival of the population as a whole. From this perspective it becomes apparent that only a minor part of the *S*. *epidermidis* population undergoes (stochastic?) RsaE expression to avoid total lysis. Taken together, the data give a first insight into the role of sRNAs as subtle players in *S*. *epidermidis* biofilm organization, and it will be an exciting future task to comprehensively establish RsaE functions in this organism, including its complete target spectrum and its role as a regulator of metabolic functions.

## Methods

### Bacterial strains and culture conditions

All bacterial strains and plasmids used in this study are listed in Table 1 and Table 2. *Staphylococcus* strains were cultivated in Trypticase Soy Broth (TSB; BD BBL) at 37°C with a 1:5 medium to flask ratio, while *Escherichia coli* was cultivated in Luria-Bertani broth (LB). TSB and LB agar plates contained 1.5% agar. When needed for plasmid maintenance, ampicillin (Amp; 100 μg/ml; in *E*. *coli*) or chloramphenicol (Cm; 30 μg/ml; in *Staphylococcus*) was added to the growth medium. Anhydrotetracycline (ATc; 25–100 ng/ml was added to induce pCG248 vector derived gene expression.

**Table 1:** List of bacterial strains.

Strain	Properties	Reference
*E*. *coli* DC10B	*E*. *coli* DH10B Δ*dcm* (cytosine methylase deficient)	[58]
*S*. *aureus* PS187 Δ*hsdR* Δ*sauUSI*	ST395, deficient in restriction system type IV + I, markerless mutant, host for phage Φ187	[59]
*S*. *epidermidis* ATCC12228	biofilm-negative, *ica*-negative	[60]
*S*. *epidermidis* RP62A	Clinical isolate, biofilm-positive reference strain, *ica*-positive	[61]
*S*. *epidermidis* PS2	blood culture isolate, biofilm-positive (protein), *ica*-positive	[17]
*S*. *epidermidis* PS10	blood culture isolate, biofilm-positive (PIA), *ica*-positive	[17]
*S*. *epidermidis* O-47	Clinical isolate, biofilm-positive, *ica*-positive	[6]
*S*. *epidermidis* 567	Clinical isolate, inducible biofilm-former, *ica*-positive	[43]
*E*. *coli* DC10B pGEM_*lrgA*	*E*. *coli* DC10B carrying pGEM T Easy with *lrgA*	this study
*E*. *coli* DC10B pGEM_mut*lrgA*	*E*. *coli* DC10B carrying pGEM T Easy with *lrgA* mutated in 6 bp after SDM (mut*lrgA*)	this study
*E*. *coli* DC10B pGEM_5’-UTR-*icaR*	*E*. *coli* DC10B carrying pGEM T Easy with 5’-UTR-*icaR*	this study
*E*. *coli* DC10B pGEM_5’-UTR-*icaR*-RBSmut	*E*. *coli* DC10B carrying pGEM T Easy with 5’-UTR-*icaR* mutated in 4 bp after SDM (5’-UTR-*icaR*-RBSmut)	this study
*S*. *epidermidis* PS10 p_(P_*rsaE*_*cfp*)	*S*. *epidermidis* PS10 carrying vector p_(P_*rsaE*_*cfp*)	this study
*S*. *epidermidis* PS10 pCerulean	*S*. *epidermidis* PS10 carrying vector pCerulean	this study
*S*. *epidermidis* PS2 p_(P_*cidA*_*cfp*/P_*lrgA*_*yfp*)	*S*. *epidermidis* PS2 carrying vector p_(P_*cidA*_*cfp*/P_lrgA_*yfp*)	this study
*S*. *epidermidis* PS2 pCG248	*S*. *epidermidis* PS2 carrying empty vector control pCG248	this study
*S*. *epidermidis* PS2 pCG248_*rsaE*	*S*. *epidermidis* PS2 carrying vector pCG248_*rsaE*	this study
*S*. *epidermidis* PS10 pCG248_*rsaE*	*S*. *epidermidis* PS10 carrying vector pCG248_*rsaE*	this study
*S*. *epidermidis* O-47 pCG248_*rsaE*	*S*. *epidermidis* O-47 carrying vector pCG248_*rsaE*	this study
*S*. *epidermidis* 567 pCG248_*rsaE*	*S*. *epidermidis* 567 carrying vector pCG248_*rsaE*	this study
*S*. *epidermidis* PS10Δ*rsaE*	Chromosomal markerless deletion mutant of *rsaE* in *S*. *epidermidis* PS10	this study
S. epidermidis PS10Δ*rsaE* pCG248_*rsaE*	*S*. *epidermidis* PS10Δ*rsaE* carrying vector pCG248_*rsaE*	this study
S. epidermidis PS10Δ*rsaE* pCG248_*rsaE*_mutated	*S*. *epidermidis* PS10Δ*rsaE* carrying vector pCG248_*rsaE*_mutated	this study
*S*. *epidermidis* O-47Δ*rsaE*	Chromosomal markerless deletion mutant of *rsaE* in *S*. *epidermidis* O-47	this study
*S*. *epidermidis* O-47Δ*rsaE* pCG248_*rsaE*	*S*. *epidermidis* O-47Δ*rsaE* carrying vector pCG248_*rsaE*	this study

**Table 2:** List of plasmids.

Plasmid	Properties	Reference
pGEM-T Easy Vector System I	ori pBR322, 3´-T overhang, system for direct cloning of PCR products	Promega
pCG248	ori ColE1, ori pT181, P_xyl/tet_ controlled gene expression, ATc inducible	[24]
pCerulean	pALC2084 expressing codon-improved Cerulean (*cfp*), ATc inducible	[62]
pKM003	integration vector in *Bacillus*; used as source for *yfp* gene	[63]
pGEM_*lrgA*	IVT template for *lrgA*, used for SDM	this study
pGEM_mut*lrgA*	IVT template for mut*lrgA*	this study
pGEM_5’-UTR-*icaR*	IVT template for 5’-UTR-*icaR*, used for SDM	this study
pGEM_5’-UTR-*icaR*-RBSmut	IVT template for 5’-UTR-*icaR*-RBSmut	this study
pCG248_*rsaE*	*rsaE* gene under control of p_tet_ promoter, ATc inducible	this study
p_(P_*rsaE*_*cfp*)	Gene *cfp* (Cerulean) under control of *rsaE* promoter	this study
p_(P_*tet*_*cfp*)	Gene *cfp* (Cerulean) under control of p_tet_ promoter, ATc inducible	this study
p_(P_*cidA*_*cfp*/P_*lrgA*_*yfp*)	Gene *cfp* under control of *cidA* promoter and gene *yfp* under control of *lrgA* promoter	this study
pCG248_*rsaE*_ mutated	*rsaE* gene with C-rich motifs 1–3 mutated to A's under control of p_tet_ promoter, ATc inducible	this study

### Preparation of total RNA from bacterial cells

Samples from bacterial cultures were mixed with 0.2X vol. STOP mix solution (95% (v/v) ethanol/ 5% (v/v) aqua-phenol, cooled to -20°C) for immediate RNA stabilization. Cells were disrupted mechanically using Lysing Matrix E glass bead tubes and the FastPrep Instrument (MP Biomedicals) and total RNA was extracted using the hot phenol technique. In brief, the supernatant of the disrupted cells was mixed with SDS (1%), sodium acetate (pH 5.5, 0.1 M) and phenol (1:1) and incubated at 64°C in a water bath for 10 min. The aqueous phase was then transferred to PLG tubes (phase-lock gel tubes, cat. no. 2302830, 5 Prime) and after chloroform extraction (1:1), the aqueous phase was again transferred to fresh tubes and the RNA precipitated with 4X vol. ethanol/3 M sodium acetate pH 6.5 (30:1 mix) at -20°C overnight. Pelleted RNA was washed with 70% ethanol and finally solved in RNase-free water. RNA was stored at -80°C, quantity was measured using the NanoDrop system (Thermo Scientific, ND-2000), while RNA quality was assessed with the Agilent 2100 Bioanalyzer (Agilent Technologies) prior to downstream applications.

### Northern blot analysis

6% or 10% (as indicated) polyacrylamide gels, containing 7 M urea for denaturing conditions, were used for electrophoretic separation of total RNA (10 μg per sample) according to standard procedures. After gel electrophoresis, RNA was blotted onto a nylon membrane (GE Healthcare, Hybond-XL) using a wet blotting chamber. RNA was cross-linked to the membrane by UV light and subsequently used for hybridization with sequence specific, radioactively labeled probes. Oligonucleotides (ssDNA) listed in S2 Table were 5’-endlabeled using T4 polynucleotide kinase (PNK) and [γ^32^P]-ATP (6000 Ci/mmol, 10 μCi/μl). Radioactive signals were detected by storage phosphor screens (BAS-IP SR 2040 E) and the Typhoon FLA 7000 laser scanner (GE Healthcare).

### Rifampicin RNA stability assay

Bacteria from overnight cultures were diluted in 250 ml flasks in 50 ml TSB medium to an initial optical density at 600 nm (OD_600_) of 0.05 and grown with shaking at 220 rpm at 37°C to an OD_600_ of 4.5. Then rifampicin was added to the cultures at a final concentration of 100 μg/ ml. Before (t0) and after 1, 2, 4, 8, 16 and 32 minutes of rifampicin exposure bacteria were sampled, RNA was isolated and Northern blot analyses were performed as described above.

### In vitro transcription (IVT) and gel shift assay (EMSA)

Target RNAs for gel shift assays were *in vitro* transcribed using the MEGAscript Kit (Thermo Scientific) from templates generated by PCR using oligonucleotides listed in S2 Table as primers. The length of the resulting transcripts are also shown in S2 Table. RNA was purified by phenol/chloroform/isoamylalcohol extraction P:C:I (25:24:1) and ethanol precipitation. RsaE and RsaEp were radioactively labeled during IVT by addition of [α^32^P]-UTP (800 Ci/mmol, 20 μCi/μl) and non-incorporated nucleotides were removed by Illustra MicroSpin G-25 columns (GE Healthcare). Following electrophoresis on a denaturing 6% PAA/ 7 M urea gel, the bands corresponding in size to RsaE and RsaEp, respectively, were cut-out of the gel and the RNA eluted overnight (at 8°C in elution buffer: 0.1 M sodium acetate, 0.1% SDS, 10 mM EDTA pH 8.0). RNA was further purified by P:C:I (25:24:1) extraction and precipitated by ethanol at -20°C. For each EMSA, target RNAs and labeled RsaE/RsaEp were first denatured at 96°C for 2 min, cooled on ice and then mixed with 2X TMN buffer (40 mM Tris pH 7.5, 20 mM MgCl_2_, 300 mM NaCl) before renaturing separately at room temperature for 10 min. Now, labeled RsaE/RsaEp was added to the tubes containing increasing amounts of target RNA and incubated together at 37°C for 15 min before directly being loaded onto a running native 6% PAA gel after addition of native loading dye (50% glycerol, 0.2% bromophenol blue, 0.2% xylene cyanol, 0.5X TBE). After electrophoresis, gels were vacuum dried and radioactive signals detected by storage phosphor screens and the Typhoon FLA 7000 laser scanner. For competition EMSAs, the target RNA was first mixed with either increasing amounts of an antisense-RsaE oligo or with excess amounts of yeast tRNA before labeled RsaE/RsaEp was added. For competition EMSA with unlabeled RsaE, labeled RsaE was first mixed with increasing amounts of unlabeled RsaE or excess amounts of yeast tRNAs before the target RNA was added.

### Biofilm assays

Biofilm formation was tested on 96-well, polystyrene tissue culture plates (Greiner Bio-One, Cellstar, F-form) as described previously [64], using Trypticase Soy Broth (TSB; BD BBL) as growth medium. *S*. *epidermidis* RP62A and *S*. *epidermidis* ATCC12228 were used as positive and negative controls, respectively. In brief, bacterial overnight cultures (ONCs) were freshly diluted to OD_600_ of 0.05 and 200 μl filled in each well (4 wells per strain). Four tissue culture plates were set up in parallel and incubated at 37°C for 18 h. The bacteria were then discarded and adherent cells washed 3x with 1X PBS buffer before the remaining cells were fixed at 65°C (plate 1). To determine whether the biofilm was mediated by PIA production or by proteins, biofilms were either (plate 2) treated with sodium periodate (Sigma; 40 mM NaIO_4_ for 24 h at 4°C) or (plate 3) proteinase K (Merck; 1 mg/ml for 4 h at 37°C) and afterwards washed again with 1X PBS and heat fixed. Read-out was conducted via crystal violet staining and determination of absorption at 492 nm (ELISA plate reader, Multiskan Ascent). To determine the eDNA content (plate 4) biofilms were stained with Ethidium Homodimer III (1X dead staining solution from the PromoKine Bacteria Live/Dead Staining Kit, PromoCell) for 30 min, washed again with 1X PBS and fluorescence directly measured at 535/ 595 nm (TECAN infinite 200Pro).

### Confocal laser scanning microscopy (CLSM)

ONC of *S*. *epidermidis* PS10 p_(P_*rsaE*_*cfp*) was freshly diluted in TSB (+Cm 20 μg/ml) to OD_600_ of 0.05. 800 μl of the bacterial suspension were filled into a cell culture dish (μ-Dish 35 mm, low wall, uncoated, hydrophobic, sterilized; Ibidi) and 8 μl of 100X dead staining (EthD-III, Ethidium Homodimer III; from PromoKine Bacteria Live/Dead Staining Kit, PromoCell) was added to stain extracellular DNA (eDNA) as well as dead cells during biofilm growth. The culture dish was set up at the microscope stage (warmed to 30°C) and the growing cells were scanned in 1 μm steps (bottom to top layer) every 30 min for 19 h. The next day, the suspension was carefully pipetted off and adherent cells were washed by adding 800 μl 1X PBS buffer to the edge of the culture dish. To stain for PIA, 200 μl of 5 μg/ml Alexa Fluor 488 wheatgerm agglutinin (binding N-acetylglucosaminyl residues; Life technologies) was added and incubated for 10 min. The stain was washed off by adding 800 μl 1X PBS (twice) and the biofilm structure scanned directly at the confocal laser scanning microscope (CLSM; Leica Microsystems GmbH, Leica TCS SP5). Construct *S*. *epidermidis* PS2 p_(P_*cidA*_*cfp*/P_lrgA_*yfp*) was set up accordingly in TSB (with Cm 20 μg/ml and 1X dead stain EthD-III) at OD_600_ of 0.05 in a cell culture dish at the microscope stage (warmed to 30°C) and growing cells were scanned in 0.5 μm steps (bottom to top layer) every 30 min for 19 h. Alternatively, *S*. *epidermidis* PS10 p_(P_*rsaE*_*cfp*) was set up in a similar manner in two cell culture dishes and grown at 37°C for 10 h or 20 h. Then adherent cells were washed with 800 μl 1X PBS and stained for eDNA (0.5X dead stain EthD-III for 20 min) followed by PIA staining with Alexa Fluor 488 wheatgerm agglutinin (5 μg/ml for 10 min) and washed thrice with 800 μl 1X PBS before microscopy. CSLM settings were as follows: Object lens used was HCX PL APO CS 63.0x1.40 OIL UV with zoom factor 1.2 or 2; sequential scans were usually done at 200 Hz (1024x512 pixels); for EthD-III (eDNA) laser 561 nm excitation (at 10–13%) was used with detection range 600 nm– 680 nm (HyD gain at 10–25%); for Cerulean (*rsaE* promoter activity) argon laser wavelength at 458 nm (at 23%) and detection range from 460 nm– 510 nm (HyD gain at 50%) was used. WGA-Oregon Green 488 (for PIA detection) was scanned with excitation by argon laser at 488 nm (at 23%) and detection range of 520 nm– 580 nm (HyD gain at 50%). With construct *S*. *epidermidis* PS2 p_(P_*cidA*_*cfp*/P_lrgA_*yfp*) settings for eDNA (EthD-III) detection were similar; Cerulean (*cidA* promoter activity) was excited at 458 nm (at 33%) and detected from 460 nm– 510 nm (HyD gain at 75%); YFP (*lrgA* promoter activity) was excited with argon laser at wavelength 514 nm (at 9%) and detected in range 520 nm– 580 nm (HyD gain at 25%).

## Supporting information

S1 Fig Differentially transcribed metabolic genes in *S*. *epidermidis* PS2 (early) and PS10 (late) isolates based on RNAseq data analysis. The scheme was generated by employing the Interactive Pathway Explorer (https://pathways.embl.de). Genes and pathways highlighted in blue are upregulated in PS2 (and downregulated in PS10), while features downregulated in PS2 (and upregulated in PS10) are indicated in red. Visualization and interactive analysis of the data set (including regulatory pathways and secondary metabolite biosynthesis genes) is enabled by following the instructions and data provided in S1 Text.
(PDF)

S2 Fig Analysis of biofilm production of *S*. *epidermidis* O-47 (pCG248_*rsaE*) by static 96-well microtiter plate biofilm assays. Plasmid pCG248_*rsaE* harbours the *rsaE* gene under the control of an anhydrotetracycline (ATc)-inducible promoter. Expression of *rsaE* was induced by increasing ATc concentrations (25 to 75 ng/ml). Total biofilm (BF) mass as well as PIA- and protein-mediated biofilm proportions were determined by sodium-periodate and proteinase K treatments, respectively, as described in Methods. Sterile TSB medium served as background control. The strong PIA-producer *S*. *epidermidis* RP62A (*ica* locus positive) as well as *S*. *epidermidis* ATCC12228 (*ica* locus negative) were used as positive and negative controls, respectively. Graphs represent results of three independent biological replicates and error bars indicate the mean with SEM (standard error of the mean).
(PDF)

S3 Fig *lrgA/RsaE* EMSA upon competition with unlabeled RsaE (A) or with an antisense-RsaE RNA oligonucleotide (B) during complex formation. *(A)* Increasing amounts of unlabeled competitor RsaE (lanes 3–7) or a 500-fold and 1000-fold excess of yeast tRNAs (lane 8+9) were mixed with 200 nM radioactively (*) labeled RsaE before addition to 200 nM *lrgA* target RNA. *(B)* 200 nM of *lrgA* target RNA was mixed with increasing amounts of antisense-RsaE RNA oligonucleotide (lanes 3–7) or a 500-fold excess of yeast tRNAs (lane 8) prior to addition of 200 nM radioactively (*) labeled RsaE to the samples. *(C)* EMSAs of radioactively (*) labeled RsaE with increasing amounts of *lrgA* target RNA with either *S*. *epidermidis* sequence (left panel) or *S*. *aureus* sequence (right panel). The nucleotide sequence of RsaE is conserved between both species. *(D)* Nucleotide sequence comparison of the 5’ UTRs of *S*. *aureus lrgA* (top) and *S*. *epidermidis lrgA* (bottom). The interaction site of *S*. *epidermidis lrgA* with RsaE is highlighted in red. Ribosomal binding sites (RBS) are marked in bold and start codons are underlined.
(PDF)

S4 Fig EMSAs using increasing amounts of *sucD* target RNA and radioactively labeled (*) full-length (RsaE, left panel) or processed RsaE (RsaEp, right panel) as binding partners. Filled triangles indicate labeled unbound RsaE species and open triangles mark RsaE/target complexes.
(PDF)

S5 Fig (*A*) Secondary structures of full-length and processed RsaE species according to MFold-4.7-based predictions [16]. Red boxes highlight positions of the C-rich motifs within the RsaE molecules. Position of the antisense-RsaE RNA oligonucleotide is underlined. (*B*) IntaRNA predictions of full-length (top) and processed (bottom) RsaE interaction with *sucC* mRNA [17]. The RsaE C-rich motifs and the *sucC* ribosomal binding sites (RBS) are highlighted by red and by grey boxes, respectively.
(PDF)

S6 Fig RsaE expression and correlation with biofilm production and eDNA release in different *S*. *epidermidis* strains. (A) Quantification of *rsaE* transcript by qRT-PCR at the time point indicated. The graph displays relative mRNA amounts using *gyrB* expression as reference. (B) Analysis of biofilm production by static 96-well microtiter plate biofilm assays. Total biofilm (BF) mass as well as PIA- and protein-mediated biofilm proportions were determined by sodium-periodate and proteinase K treatments, respectively, as described in Methods. (C) Detection of eDNA content in *S*. *epidermidis* biofilms by Ethidium Homodimer III staining and fluorescence intensity measurements at 535/595 nm. Biofilms were grown in 96-well microtiter plates using the same strains and conditions as in (B). Graphs represent results of three independent biological replicates and error bars indicate the mean with SEM (standard error of the mean).
(PDF)

S7 Fig *rsaE* expression in planktonic culture. CLSM images of *S*. *epidermidis* PS10 p_(P_*rsaE*_CFP) during growth in liquid culture. At the indicated time points, cells were taken and subjected to CLSM imaging. Shown is an overlay of total bacterial cell mass, visualized by transmission microscopy, and *rsaE*-expressing bacterial cells, highlighted by cerulean expression (light blue).
(PDF)

S1 Video Time lapse video of CLSM live cell imaging monitoring *rsaE* promoter activity in an *S*. *epidermidis* PS10 biofilm during 20 hours of growth in chamber slides. The strain carries plasmid p_(P_*rsaE*_*cfp*) in which the *rsaE* promoter is fused to the blue-fluorescent cerulean protein gene *cfp* as a reporter. Light blue cells represent bacteria with an active *rsaE* promoter, grey cells mark the total bacterial mass. The video corresponds to Fig 2D of the main text.
(MPEG)

S2 Video Time lapse video of CLSM live cell imaging monitoring *rsaE* promoter activity (light blue) and eDNA release (red) in an *S*. *epidermidis* PS10 biofilm during 20 hours of growth in chamber slides. Same video as S1 Video, but without transmission microscopy. Instead, eDNA is visualized in red by Ethidium Homodimer II staining with light blue cells representing bacteria with an active *rsaE* promoter.
(MPEG)

S1 Table dRNA-seq transcription profiling data of *S*. *epidermidis* PS2 and PS10. Complete list of (differentially expressed) genes. (XLSX)

S2 Table List of oligonucleotides used in this study. (PDF)

S3 Table List of RsaE target mRNA predictions by IntaRNA. (PDF)

S1 Text Supporting information. Methods.
(DOCX)
