# Supplementary material for: The small non-coding RNA RsaE influences extracellular matrix composition in Staphylococcus epidermidis biofilm communities
Source: PLoS Pathog. 2019 Mar 14;15(3):e1007618. doi: 10.1371/journal.ppat.1007618 (PMC6435200; doi:10.1371/journal.ppat.1007618)
Supplement: S1 Fig — The scheme was generated by employing the Interactive Pathway Explorer (https://pathways.embl.de). Genes and pathways highlighted in blue are upregulated in PS2 (and downregulated in PS10), while features downregulated in PS2 (and upregulated in PS10) are indicated in red. Visualization and interactive analysis of the data set (including regulatory pathways and secondary metabolite biosynthesis genes) is enabled by following the instructions and data provided in S1 Text. (PDF) [file ppat.1007618.s001.pdf]

Figure S1

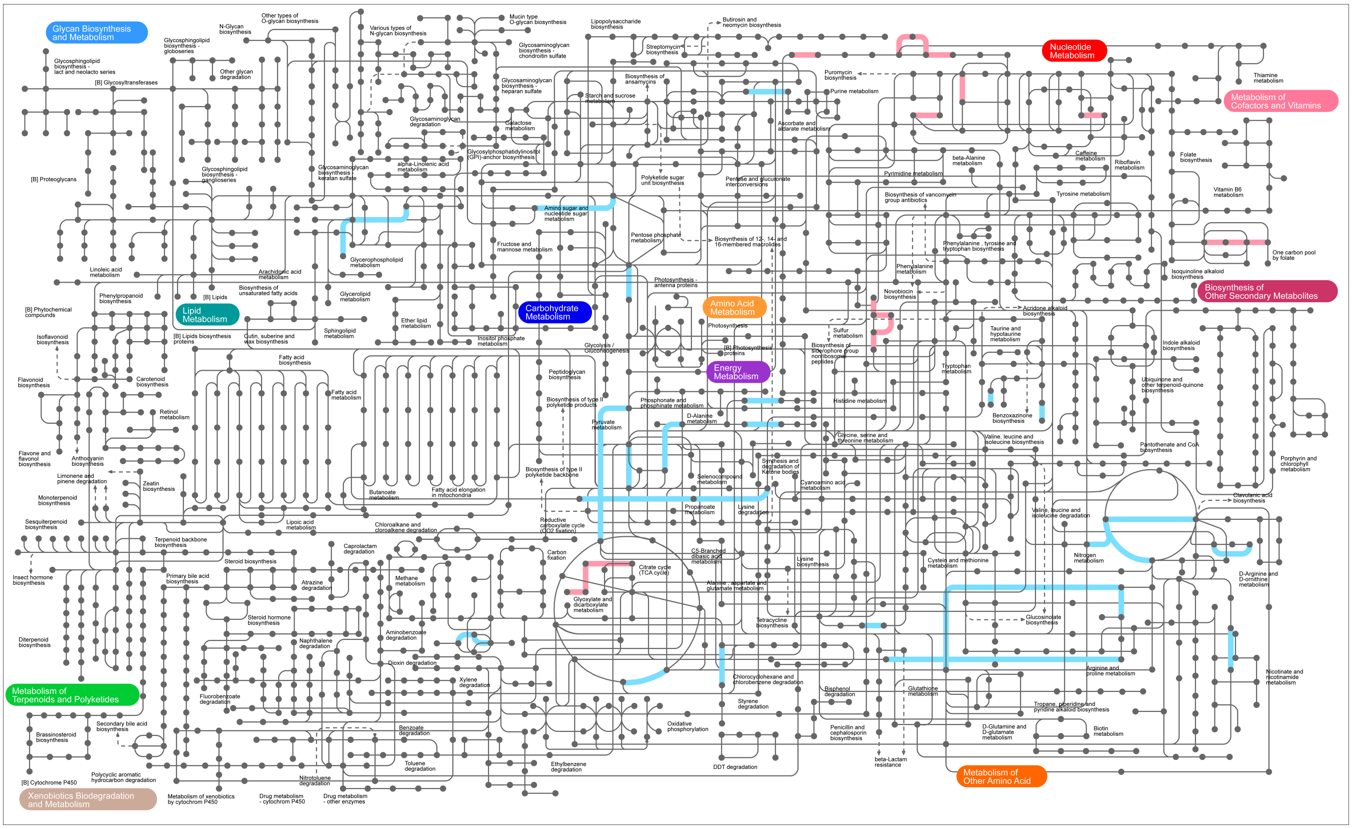

S1 Figure: Differentially transcribed metabolic genes in *S. epidermidis* PS2 (early) and PS10 (late) isolates based on RNAseq data analysis. The scheme was generated by employing the Interactive Pathway Explorer (https://pathways.embl.de). Genes and pathways highlighted in blue are upregulated in PS2 (and downregulated in PS10), while features downregulated in PS2 (and upregulated in PS10) are indicated in red. Visualization and interactive analysis of the data set (including regulatory pathways and secondary metabolite biosynthesis genes) is enabled by following the instructions and data provided in S1 Text.
