# Supplementary material for: The small non-coding RNA RsaE influences extracellular matrix composition in Staphylococcus epidermidis biofilm communities
Source: PLoS Pathog. 2019 Mar 14;15(3):e1007618. doi: 10.1371/journal.ppat.1007618 (PMC6435200; doi:10.1371/journal.ppat.1007618)
Supplement: S2 Fig — Plasmid pCG248_rsaE harbours the rsaE gene under the control of an anhydrotetracycline (ATc)-inducible promoter. Expression of rsaE was induced by increasing ATc concentrations (25 to 75 ng/ml). Total biofilm (BF) mass as well as PIA- and protein-mediated biofilm proportions were determined by sodium-periodate and proteinase K treatments, respectively, as described in Methods. Sterile TSB medium served as background control. The strong PIA-producer S. epidermidis RP62A (ica locus positive) as well as S. epidermidis ATCC12228 (ica locus negative) were used as positive and negative controls, respectively. Graphs represent results of three independent biological replicates and error bars indicate the mean with SEM (standard error of the mean). (PDF) [file ppat.1007618.s002.pdf]

Figure S2

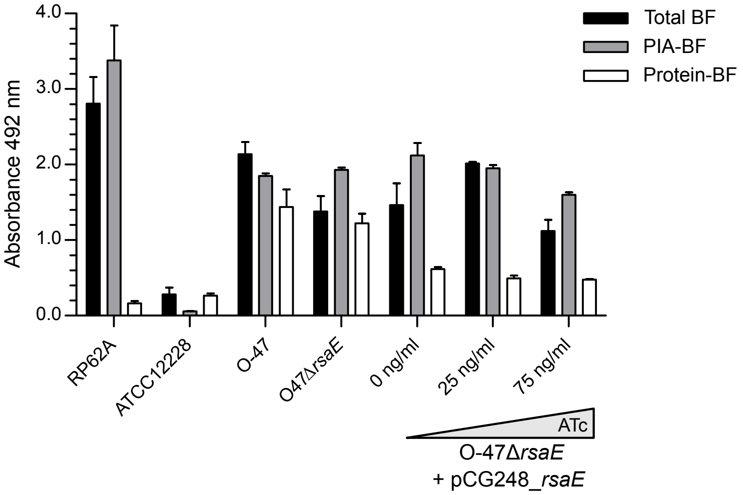

S2 Figure: Analysis of biofilm production of *S. epidermidis* O-47 and O-47 Δ*rsaE* (pCG248_*rsaE*) by static 96-well microtiter plate biofilm assays. Plasmid pCG248_*rsaE* harbours the *rsaE* gene under the control of an anhydrotetracycline (ATc)-inducible promoter. Expression of *rsaE* was induced by increasing ATc concentrations (25 to 75 ng/ml). Total biofilm (BF) mass as well as PIA- and protein-mediated biofilm proportions were determined by sodium-periodate and proteinase K treatments, respectively, as described in Methods. The strong PIA-producer *S. epidermidis* RP62A (*ica* locus positive) as well as *S. epidermidis* ATCC12228 (*ica* locus negative) were used as positive and negative controls, respectively. Graphs represent results of three independent biological replicates and error bars indicate the mean with SEM (standard error of the mean).
