# Supplementary material for: The small non-coding RNA RsaE influences extracellular matrix composition in Staphylococcus epidermidis biofilm communities
Source: PLoS Pathog. 2019 Mar 14;15(3):e1007618. doi: 10.1371/journal.ppat.1007618 (PMC6435200; doi:10.1371/journal.ppat.1007618)
Supplement: S3 Fig — lrgA/RsaE EMSA upon competition with unlabeled RsaE (A) or with an antisense-RsaE RNA oligonucleotide (B) during complex formation. (A) Increasing amounts of unlabeled competitor RsaE (lanes 3–7) or a 500-fold and 1000-fold excess of yeast tRNAs (lane 8+9) were mixed with 200 nM radioactively (*) labeled RsaE before addition to 200 nM lrgA target RNA. (B) 200 nM of lrgA target RNA was mixed with increasing amounts of antisense-RsaE RNA oligonucleotide (lanes 3–7) or a 500-fold excess of yeast tRNAs (lane 8) prior to addition of 200 nM radioactively (*) labeled RsaE to the samples. (C) EMSAs of radioactively (*) labeled RsaE with increasing amounts of lrgA target RNA with either S. epidermidis sequence (left panel) or S. aureus sequence (right panel). The nucleotide sequence of RsaE is conserved between both species. (D) Nucleotide sequence comparison of the 5’ UTRs of S. aureus lrgA (top) and S. epidermidis lrgA (bottom). The interaction site of S. epidermidis lrgA with RsaE is highlighted in red. Ribosomal binding sites (RBS) are marked in bold and start codons are underlined. (PDF) [file ppat.1007618.s003.pdf]

Figure S3

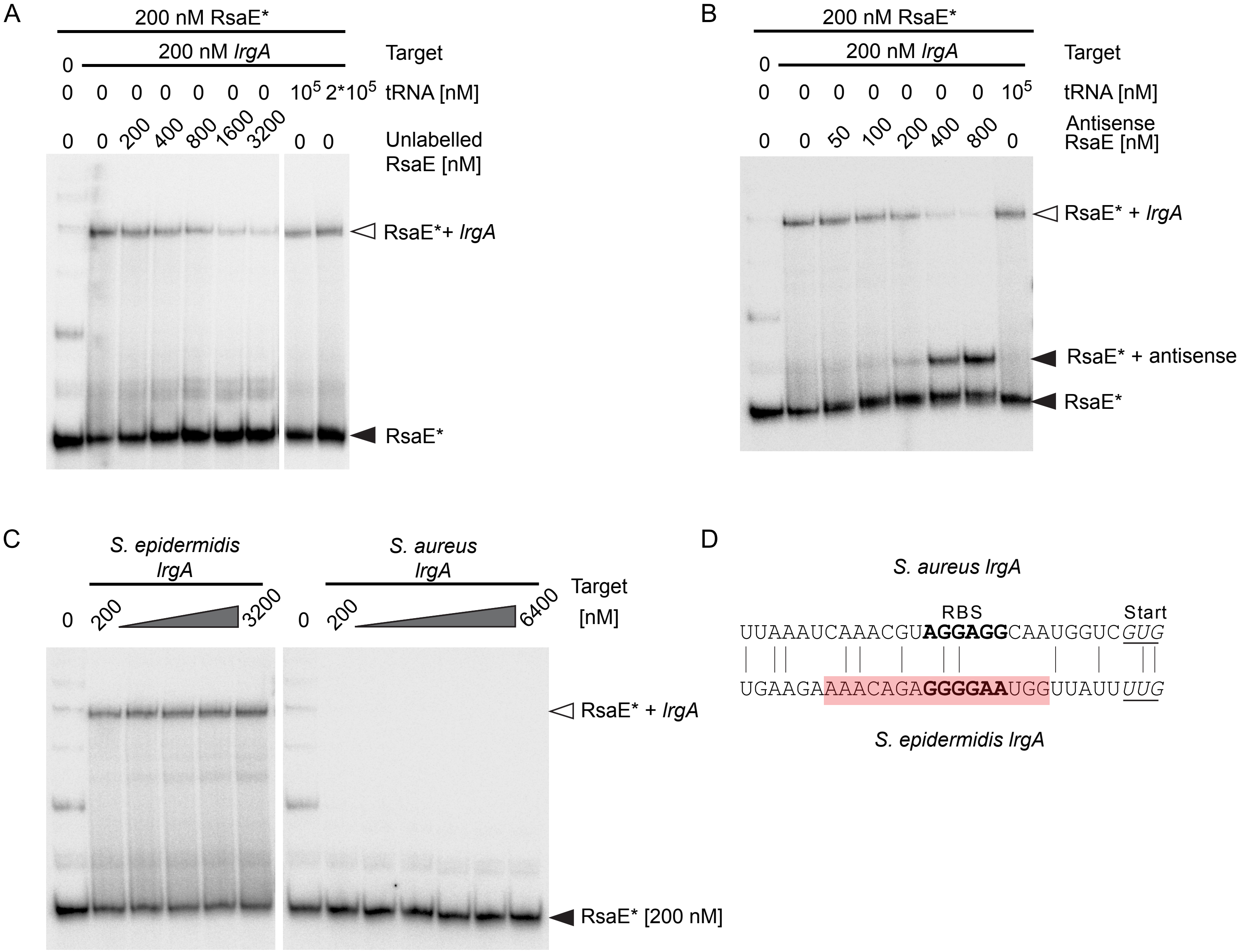

S3 Figure: *lrgA/rsaE* EMSA upon competition with unlabeled RsaE (A) or with an antisense-RsaE RNA oligonucleotide (B) during complex formation. (A) Increasing amounts of unlabeled competitor RsaE (lanes 3-7) or a 500-fold and 1000-fold excess of yeast tRNAs (lane 8+9) were mixed with 200 nM radioactively (*) labeled RsaE before addition to 200 nM *lrgA* target RNA. (B) 200 nM of *lrgA* target RNA was mixed with increasing amounts of antisense-RsaE RNA oligonucleotide (lanes 3-7) or a 500-fold excess of yeast tRNAs (lane 8) prior to addition of 200 nM radioactively (*) labeled RsaE to the samples. (C) EMSAs of radioactively (*) labeled RsaE with increasing amounts of *lrgA* target RNA with either *S. epidermidis* sequence (left panel) or *S. aureus* sequence (right panel). The nucleotide sequence of RsaE is conserved between both species. (D) Nucleotide sequence comparison of the 5' UTRs of *S. aureus lrgA* (top) and *S. epidermidis lrgA* (bottom). The interaction site of *S. epidermidislrgA* with RsaE is highlighted in red. Ribosomal binding sites (RBS) are marked in bold and start codons are underlined.
