# Supplementary material for: The small non-coding RNA RsaE influences extracellular matrix composition in Staphylococcus epidermidis biofilm communities
Source: PLoS Pathog. 2019 Mar 14;15(3):e1007618. doi: 10.1371/journal.ppat.1007618 (PMC6435200; doi:10.1371/journal.ppat.1007618)
Supplement: S4 Fig — EMSAs using increasing amounts of sucD target RNA and radioactively labeled (*) full-length (RsaE, left panel) or processed RsaE (RsaEp, right panel) as binding partners. Filled triangles indicate labeled unbound RsaE species and open triangles mark RsaE/target complexes. (PDF) [file ppat.1007618.s004.pdf]

Figure S4

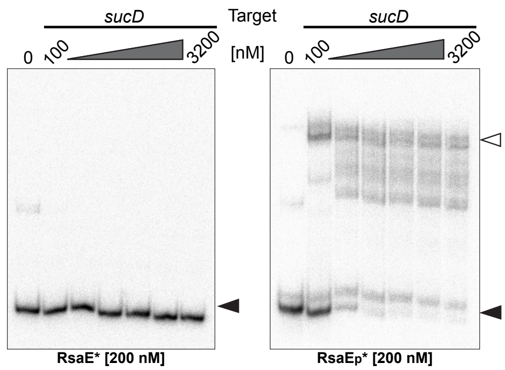

S4 Figure: EMSAs using increasing amounts of *sucD* target RNA and radioactively labeled (*) full-length (RsaE, left panel) or processed RsaE (RsaEp, right panel) as binding partners. Filled triangles indicate labeled unbound RsaE species and open triangles mark RsaE/target complexes.
