# Supplementary material for: The small non-coding RNA RsaE influences extracellular matrix composition in Staphylococcus epidermidis biofilm communities
Source: PLoS Pathog. 2019 Mar 14;15(3):e1007618. doi: 10.1371/journal.ppat.1007618 (PMC6435200; doi:10.1371/journal.ppat.1007618)
Supplement: S5 Fig — (A) Secondary structures of full-length and processed RsaE species according to MFold-4.7-based predictions [16]. Red boxes highlight positions of the C-rich motifs within the RsaE molecules. Position of the antisense-RsaE RNA oligonucleotide is underlined. (B) IntaRNA predictions of full-length (top) and processed (bottom) RsaE interaction with sucC mRNA [17]. The RsaE C-rich motifs and the sucC ribosomal binding sites (RBS) are highlighted by red and by grey boxes, respectively. (PDF) [file ppat.1007618.s005.pdf]

Figure S5

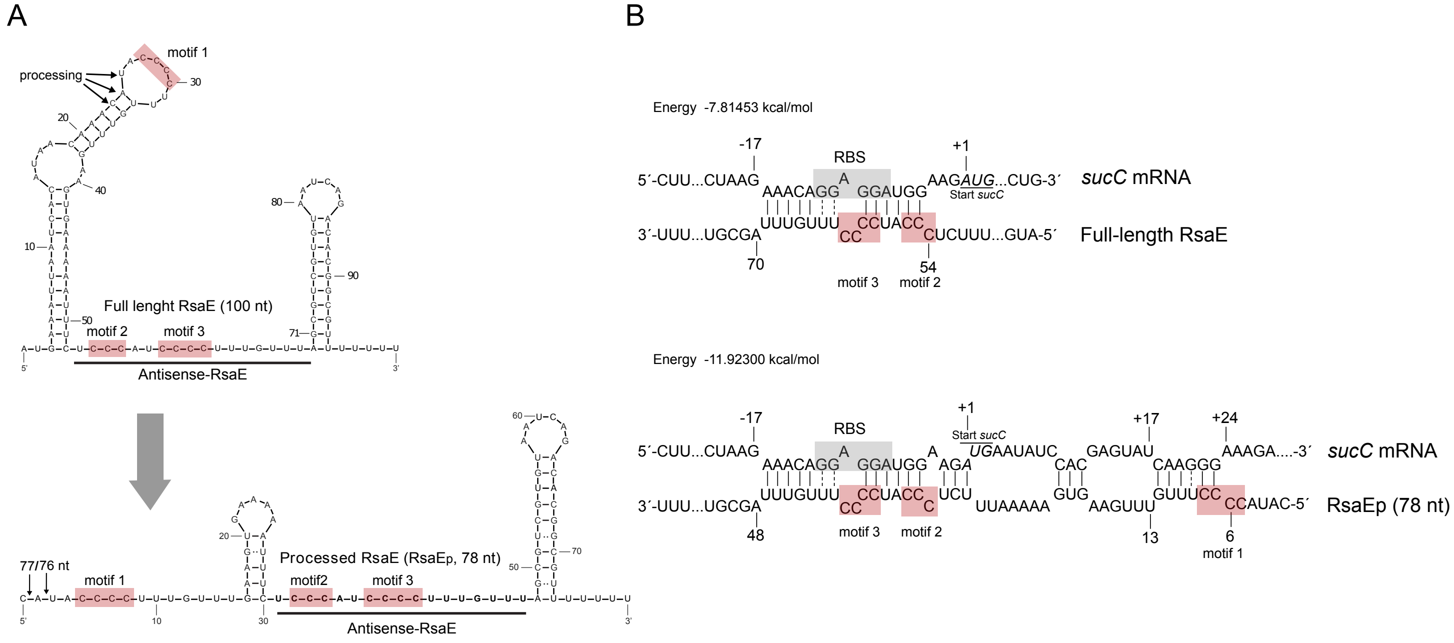

S5 Figure: (A) Secondary structures of full-length and processed RsaE species according to Mfold-4.7-based predictions [16]. Red boxes highlight positions of the C-rich motifs within the RsaE molecules. Position of the antisense-RsaE RNA oligonucleotide is underlined. (B) IntaRNA predictions of full-length (top) and processed (bottom) RsaE interaction with *sucC* mRNA [17]. The RsaE C-rich motifs and the *sucC* ribosomal binding sites (RBS) are highlighted by red and grey boxes, respectively.
