# Supplementary material for: The small non-coding RNA RsaE influences extracellular matrix composition in Staphylococcus epidermidis biofilm communities
Source: PLoS Pathog. 2019 Mar 14;15(3):e1007618. doi: 10.1371/journal.ppat.1007618 (PMC6435200; doi:10.1371/journal.ppat.1007618)
Supplement: S6 Fig — (A) Quantification of rsaE transcript by qRT-PCR at the time point indicated. The graph displays relative mRNA amounts using gyrB expression as reference. (B) Analysis of biofilm production by static 96-well microtiter plate biofilm assays. Total biofilm (BF) mass as well as PIA- and protein-mediated biofilm proportions were determined by sodium-periodate and proteinase K treatments, respectively, as described in Methods. (C) Detection of eDNA content in S. epidermidis biofilms by Ethidium Homodimer III staining and fluorescence intensity measurements at 535/595 nm. Biofilms were grown in 96-well microtiter plates using the same strains and conditions as in (B). Graphs represent results of three independent biological replicates and error bars indicate the mean with SEM (standard error of the mean). (PDF) [file ppat.1007618.s006.pdf]

Figure S6

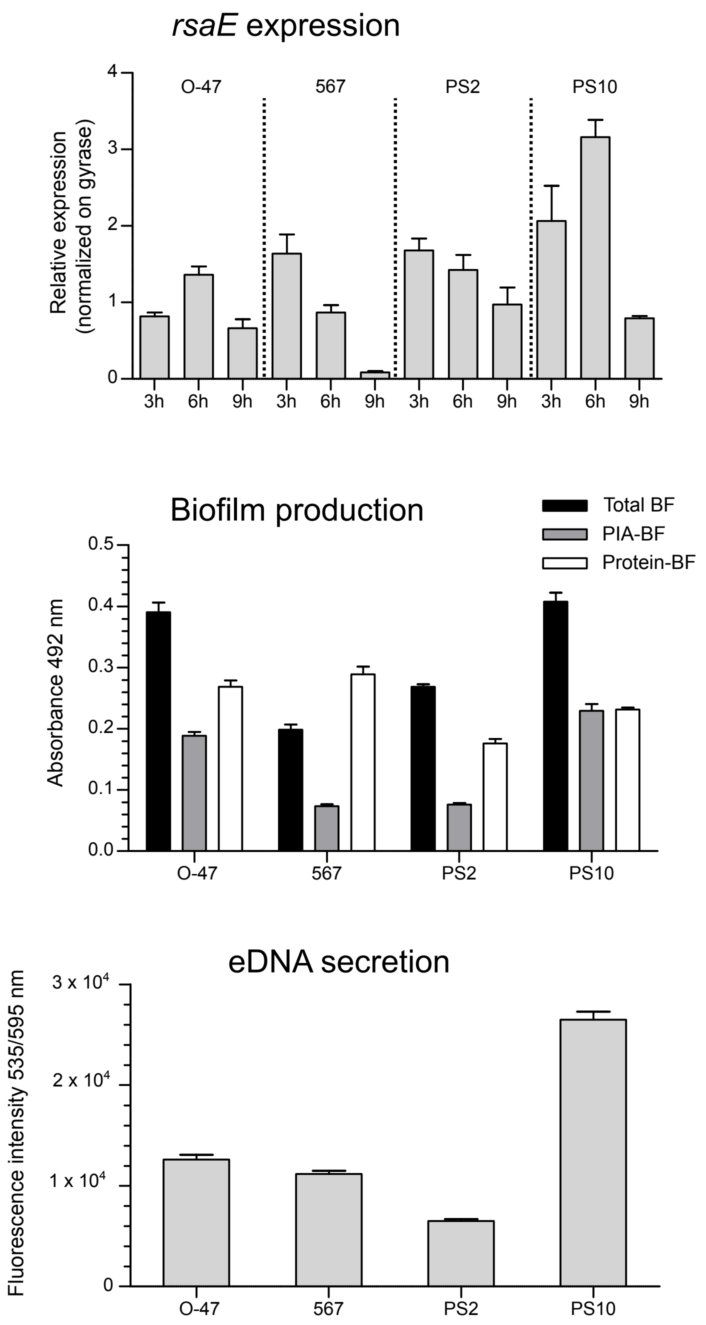

S6 Figure: RsaE expression and correlation with biofilm production and eDNA release in different *S. epidermidis* strains. (A) Quantification of *rsaE* transcript by qRT-PCR at the time point indicated. The graph displays relative mRNA amounts using *gyrB* expression as reference. (B) Analysis of biofilm production by static 96-well microtiter plate biofilm assays. Total biofilm (BF) mass as well as PIA- and protein-mediated biofilm proportions were determined by sodium-periodate and proteinase K treatments, respectively, as described in Methods. (C) Detection of eDNA content in *S. epidermidis* biofilms by Ethidium Homodimer III staining and fluorescence intensity measurements at 535/595 nm. Biofilms were grown in 96-well microtiter plates using the same strains and conditions as in (B).

Graphs represent results of three independent biological replicates and error bars indicate the mean with SEM (standard error of the mean).
