# Supplementary material for: The small non-coding RNA RsaE influences extracellular matrix composition in Staphylococcus epidermidis biofilm communities
Source: PLoS Pathog. 2019 Mar 14;15(3):e1007618. doi: 10.1371/journal.ppat.1007618 (PMC6435200; doi:10.1371/journal.ppat.1007618)
Supplement: S7 Fig — At the indicated time points, cells were taken and subjected to CLSM imaging. Shown is an overlay of total bacterial cell mass, visualized by transmission microscopy, and rsaE-expressing bacterial cells, highlighted by cerulean expression (light blue). (PDF) [file ppat.1007618.s007.pdf]

Figure S7

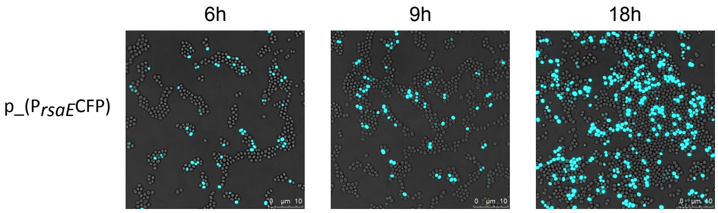

S7 Figure: *rsaE* expression in planktonic culture. CLSM images of *S. epidermidis* PS10 p_(P$_{rsaE}$CFP) during growth in liquid culture. At the indicated time point, cells were taken and subjected to CLSM imaging. Shown is an overlay of total bacterial cell mass, visualized by transmission microscopy, and *rsaE*-expressing bacterial cells, highlighted by cerulean expression (light blue).
