# Supplementary material for: The small non-coding RNA RsaE influences extracellular matrix composition in Staphylococcus epidermidis biofilm communities
Source: PLoS Pathog. 2019 Mar 14;15(3):e1007618. doi: 10.1371/journal.ppat.1007618 (PMC6435200; doi:10.1371/journal.ppat.1007618)
Supplement: S2 Table — (PDF) [file ppat.1007618.s011.pdf]

S2 Table: Lists of oligonucleotides used in this study

Primer name	5'-3' sequence	Used for
**Cloning of inducible RsaE expression**		
RsaE_BglII_for	AGTT*AGATCT*GAACATGAAATTAATCACATAACAAAC	cloning of *rsaE* gene into vector pCG248 via *Xma*I/*Bgl*II sites
RsaE_XmaI_rev	AATT*CCCGGG*CACAATAATGTATATATGATGTATA	
pCG248_out_5'p	ATTTTAATTATACTCTATCAATGATAGAGTGTCAA	outward PCR to remove 5' transcriptional overhang from RsaE insert
rsaE_out_5'p	GAAATTAATCACATAACAAACATACCCCTTTG	
pCG248_out2_5'p	CCCGGGTACCGAGCTCG	outward PCR to create empty vector control
pCG248-MCS_rev	TCTCGAAAATAATAGAGGGAAAATCAG	sequencing of constructs
pCG248-MCS_rev2	AGCTAACACATAGCCCATTCCAACCA	
pRAB11-MCS_for	GCAGCTCTAATGCGCTGTTAATCAC	
**Generation of IVT templates**		
F_RsaE_T7	TAATACGACTCACTATAGGGATGAAATTAATCACATAACAAAC	IVT of RsaE (100 nt)
R_RsaE	AAAAAAACGCCGTGTCTGATTACACGACGC	
F_RsaEp_T7	TAATACGACTCACTATAGGGTACCCCTTTGTTTGAAGT	IVT of RsaEp (76 nt)
For_LrgA_T7	TAATACGACTCACTATAGGGAGTTTCACCTTTAAAATGGTGC	IVT of *lrgA*/mut*lrgA* (SERP2026) (213 nt)
Rev_LrgA	GCAATCGTTAATGCTTGTTGAAAAATACTG	

For_CidA_T7	TAATACGACTCACTATAGGGTGCTAGCGTTTTCATAGATTTAAGG	IVT of *cidA* (SERP2117) (205 nt)
Rev_CidA	GTAATAAGCATGATAAGGGCAAGTTG	
For_SucC_T7	TAATACGACTCACTATAGGGCTTAAATTGCAATAAATACTGTC	IVT of *sucC* (SERP0813) (182 nt)
Rev_SucC	CAGCAGTAAATGCTACTCGTCCCTC	
For_SucD_T7	TAATACGACTCACTATAGGGCAGGTTTAGCTATTGAGCC	IVT of *sucD* (SERP0814) (157 nt)
Rev_SucD	GTAGACCCTGTGATACCTTGTACC	
F_icaR5'_T7	CTAATACGACTCACTATAGGGTAATATTTGTAATTTTAACTTAATTTTTCTGTA	IVT of 5'-*icaR* (SERP2292) (155 nt)
R_icaR5'	AGAAATATCATCAAGTGTAGTACCATCG	
For_Sa-LrgA_T7	TAATACGACTCACTATAGGGTAATCCTTTTTTTTATGCATTTTAC	IVT of *S. aureus lrgA* (SA0252) (239 nt)
Rev_Sa-LrgA	CTGATGCAGGCATAGGAATTGGCATAAATG	
For_LrgA_SDM	ATGGTTATTTTGGAACAAACGCATCTCG	SDM of *lrgA* to mut*lrgA* (213 nt)
Rev_LrgA_SDM	CAAAATAACCAT**aggga**CTGTTTTCTTC	
icaR5'_SDG4Umut_1	GAATAAG**tttt**AGATTTTAGAATTGAAAGATAAGA	SDM of 5'-UTR-*icaR* to 5'-UTR-*icaR*-RBSmut (157 nt)
icaR5'_SDG4Umut_2	TCT**aaaa**CTTATTCAATTTTCTAAAAATATATTACAG	
**Construction of fluorescence gene promoter fusions**		
RsaE_BamHI	ATGACT*GGATCC*TTGAATTCGCTTACAATATGAACA	amplification of *rsaE* promoter region
RsaE_BglII	CGT*TATAGA*TCTTGTTCATAATATAACACGGTAAAA	
cerul_BamHI	ATA*GGATCC*GTGATGGTAACTTCACGGTAAC	linearization of

cerul_BglII_sd	TGTA*AGATCT*ACCAGATCCTAGGAAAGGAGG	pCerulean + RBS
cerul_BglII	GCGG*AGATCT*ATGGTTTCAAAAGGTGAAGAATTATTC	linearization of pCerulean w/o RBS
cidA_BamHI	CTTG*GGATCC*GAGCGAAGTTGTTAAAGATGTGAAATAA	amplification of *cidA* promoter region + RBS
cidA_BglII	CCCGC*AGATCT*CACCCCTTTCTAAAAATGTGT	
lrgA_SalI	CCGT*GTCGAC*CATGCTTTATGAGTTTCACCTTTAA	amplification of *lrgA* promoter region + RBS
lrgA_BamHI_HindIII	*GGATCC*GTTAAACTATCA*AAGCTT*TCCCCTCTGTTTTCTTCATGG	
pCer_MCS_F	CGTTATACAAATTTTAACCCTGTTAGGAACT	sequencing of constructs
pCer_MCS_R	TGTTAATGTTGTTACTAATGTTGGCCAAG	
MF8	GGGATGTGCTGCAAGGCG	
**Oligos for Northern blot analysis**		
RsaE_1-24nt	TGTTTGTTATGTGATTAATTTCAT	detection of full-length RsaE only
RsaE_33-54nt	GAGAAATTTTTCACTTCAAACA	detection of both RsaEp and full-length RsaE
5S rDNA Oligo	CAGTCCGACTACCATCGGCG	detection of 5S rRNA
**Antisense RsaE RNA oligo for competition EMSA**		
S.e. RsaE_69-53	AAACAAAGGGGAUGGGA	Antisense RsaE RNA oligo
**Construction of *rsaE* deletion mutant**		
F_delRsaE_SalI	ATAT*GTCGAC*GAGTTACTAGGTTTAGAC	Amplification of *rsaE* flanking region
R_delRsaE_SalI	ATTA*GTCGAC*GTGAAGATACTAATCTATCAC	
RsaE_out1_5'p	GTTCATAATATAACACGGTAAAATATCC	Creation of *rsaE*

RsaE_out2_5'p	ATTTGCAAATTTTTAAAGTGAATTTAAAAATTTTTGC	deleted construct for chromosomal integration
SAP-rsaE-del2-F	TATTCCAGAAGCTGTTTACGATAAC	sequencing
SAP-rsaE-del2-R	TACTGCAAATCACATGGTAAATTCC	
**Mutation of rsaE C-rich motifs**		
RsaE_mot1C4A_out1	CATA**aaaa**TTTGTTTGAAGTGAAAAATTTCTC	Mutation of *rsaE* motif 1
RsaE_mot1C4A_out2	ACAAA**tttt**TATGTTTGTTATGTGATTAATTTC	
SAP_rsaE mot2 mut C4A for	GAAAAATTTCT**aaa**ATAAAATTTGTTTAGCGTCG	Mutation of *rsaE* motif 2
SAP_rsaE mot2 mut C4A rev	T**ttt**AGAAATTTTTCACTTCAAACAAAGGGG	
RsaEtrunc_mot3C4A_out1	CCAT**aaaa**TTTGTTTAGCGTCGTGTA	Mutation of *rsaE* motif 3
RsaEtrunc_mot3C4A_out2	CAAA**tttt**ATGGGAGAAATTTTTCACTTCA	
**qRT-PCR primers**		
Cla012	GAAATTAATCACATAACAAACATACCCCT	primers for *rsaE*
Cla013	GTGTCTGATTACACGACGCTAAACA	
gyrB-1_RT	TCTGGCGGTCTTCACGGTGTTG	primers for gyrase
gyrB-2_RT	AGGCTTGATGATAAATCGTGCCA	

Restriction enzyme sites are in italic and underlined. The T7 promoter sequence is underlined. Nucleotides exchange by site directed mutagenesis (SDM) are in small print and bold.
