# Supplementary material for: The small non-coding RNA RsaE influences extracellular matrix composition in Staphylococcus epidermidis biofilm communities
Source: PLoS Pathog. 2019 Mar 14;15(3):e1007618. doi: 10.1371/journal.ppat.1007618 (PMC6435200; doi:10.1371/journal.ppat.1007618)
Supplement: S3 Table — (PDF) [file ppat.1007618.s012.pdf]

S3 Table: List of RsaE target mRNA predictions by IntaRNA using RsaE from *S. epidermidis* as query and the *S. epidermidis* RP62A genome (NC_002976) as target sequences. The list is sorted according to energy values.

p-value	fdr value	Target	Position	Query	Position	Energy	Annotation
0.0011393	0.88564	SERP_RS12360	54 -- 70	RsaE	53 -- 69	-20.74400	Derived by automated computational analysis using gene prediction method: Protein Homology.
0.0021168	0.88564	SERP_RS08610	58 -- 70	RsaE	54 -- 67	-18.92900	TIGR01440 family protein
0.0022602	0.88564	SERP_RS03755	57 -- 87	RsaE	47 -- 69	-18.73940	Derived by automated computational analysis using gene prediction method: Protein Homology.
0.0033821	0.88564	SERP_RS10085	64 -- 75	RsaE	58 -- 69	-17.58450	antiholin-like protein LrgA
0.0057901	0.88564	SERP_RS08415	130 -- 141	RsaE	58 -- 69	-16.07090	mRNA interferase MazF
0.0059366	0.88564	SERP_RS05200	56 -- 67	RsaE	58 -- 69	-16.00130	glycerol-3-phosphate dehydrogenase (NAD(P)(+))
0.0064258	0.88564	SERP_RS06320	54 -- 69	RsaE	54 -- 69	-15.78110	membrane protein
0.0089575	0.88564	SERP_RS05790	61 -- 85	RsaE	49 -- 67	-14.86430	divalent metal cation transporter
0.0104433	0.88564	SERP_RS08560	135 -- 147	RsaE	56 -- 68	-14.44430	ATP synthase subunit gamma
0.0124966	0.88564	SERP_RS01465	62 -- 72	RsaE	56 -- 66	-13.95590	DUF2088 domain-containing protein
0.0129513	0.88564	SERP_RS12070	63 -- 71	RsaE	55 -- 63	-13.85900	type II CRISPR-associated endonuclease Cas1
0.0132963	0.88564	SERP_RS06295	56 -- 67	RsaE	58 -- 69	-13.78780	universal stress protein UspA
0.013607	0.88564	SERP_RS06790	55 -- 68	RsaE	58 -- 70	-13.72530	signal transduction protein TRAP
0.0151311	0.88564	SERP_RS05925	61 -- 68	RsaE	53 -- 60	-13.43860	cell wall amidase
0.0159223	0.88564	SERP_RS04870	58 -- 68	RsaE	58 -- 68	-13.30130	DUF1033 domain-containing protein
0.0180358	0.88564	SERP_RS02795	58 -- 69	RsaE	58 -- 69	-12.96650	ornithine aminotransferase
0.0180702	0.88564	SERP_RS09930	57 -- 70	RsaE	53 -- 67	-12.96140	sirohydrochlorin chelatase
0.0194438	0.88564	SERP_RS10290	3 -- 12	RsaE	57 -- 66	-12.76520	MFS transporter
0.0197206	0.88564	SERP_RS00365	51 -- 65	RsaE	54 -- 69	-12.72740	mechanosensitive ion channel family protein
0.020338	0.88564	SERP_RS09555	48 -- 65	RsaE	52 -- 68	-12.64500	PTS alpha-glucoside transporter subunit IIBC
0.0205608	0.88564	SERP_RS08020	57 -- 69	RsaE	58 -- 69	-12.61590	Derived by automated computational analysis using gene prediction method: Protein Homology.
0.0218725	0.88564	SERP_RS11700	81 -- 88	RsaE	58 -- 65	-12.45090	FAD:protein FMN transferase
0.0219596	0.88564	SERP_RS04105	47 -- 67	RsaE	53 -- 69	-12.44030	succinyl-CoA ligase subunit alpha
0.0222424	0.88564	SERP_RS04625	61 -- 70	RsaE	57 -- 66	-12.40620	aconitate hydratase
0.0223051	0.88564	SERP_RS10415	60 -- 82	RsaE	53 -- 69	-12.39870	xylulokinase

0.0223495	0.88564	SERP_RS12025	59 -- 66	RsaE	57 -- 64	-12.39340	Derived by automated computational analysis using gene prediction method: Protein Homology.
0.02264	0.88564	SERP_RS04455	59 -- 72	RsaE	53 -- 65	-12.35900	Derived by automated computational analysis using gene prediction method: Protein Homology.
0.0229189	0.88564	SERP_RS09785	61 -- 85	RsaE	49 -- 66	-12.32640	N-acetyltransferase
0.0232057	0.88564	SERP_RS09285	61 -- 68	RsaE	58 -- 65	-12.29330	molybdopterin converting factor subunit 1
0.0232887	0.88564	SERP_RS09525	93 -- 105	RsaE	55 -- 67	-12.28380	amino acid permease
0.0233001	0.88564	SERP_RS02205	61 -- 71	RsaE	55 -- 65	-12.28250	ComF family protein
0.0233018	0.88564	SERP_RS08095	61 -- 69	RsaE	58 -- 66	-12.28230	Derived by automated computational analysis using gene prediction method: Protein Homology.
0.0238459	0.88564	SERP_RS10110	58 -- 67	RsaE	58 -- 67	-12.22090	glycine/betaine ABC transporter ATP-binding protein
0.023863	0.88564	SERP_RS11755	65 -- 73	RsaE	57 -- 65	-12.21900	8-amino-7-oxononanoate synthase
0.0240359	0.88564	SERP_RS06220	59 -- 67	RsaE	58 -- 66	-12.19980	citrate synthase
0.0240531	0.88564	SERP_RS04125	59 -- 72	RsaE	55 -- 68	-12.19790	tyrosine recombinase XerC
0.0241029	0.88564	SERP_RS07555	92 -- 106	RsaE	55 -- 69	-12.19240	oxidoreductase
0.0241165	0.88564	SERP_RS09665	59 -- 67	RsaE	59 -- 67	-12.19090	sodium/glutamate symporter
0.0241847	0.88564	SERP_RS07850	58 -- 67	RsaE	58 -- 67	-12.18340	Derived by automated computational analysis using gene prediction method: Protein Homology.
0.0249098	0.88564	SERP_RS10625	8 -- 18	RsaE	56 -- 65	-12.10490	amino acid permease
0.0255926	0.88564	SERP_RS10235	59 -- 91	RsaE	46 -- 69	-12.03310	gluconate permease
0.0256157	0.88564	SERP_RS07400	23 -- 33	RsaE	58 -- 68	-12.03070	transposase
0.0260274	0.88564	SERP_RS06865	56 -- 84	RsaE	49 -- 72	-11.98840	sensor histidine kinase
0.0260784	0.88564	SERP_RS02165	53 -- 68	RsaE	56 -- 70	-11.98320	peptidase T
0.0264398	0.88564	SERP_RS02685	57 -- 66	RsaE	59 -- 68	-11.94670	NAD(P)/FAD-dependent oxidoreductase
0.0271831	0.88564	SERP_RS03765	24 -- 41	RsaE	48 -- 62	-11.87320	division/cell wall cluster transcriptional repressor MraZ
0.027502	0.88564	SERP_RS01570	60 -- 71	RsaE	58 -- 69	-11.84230	Na(+)/H(+) antiporter subunit B
0.0275269	0.88564	SERP_RS04930	56 -- 66	RsaE	58 -- 68	-11.83990	dihydrolipoyllysine-residue succinyltransferase component of 2-oxoglutarate dehydrogenase complex
0.0276029	0.88564	SERP_RS10845	68 -- 75	RsaE	58 -- 65	-11.83260	uroporphyrinogen-III C-methyltransferase
0.0277219	0.88564	SERP_RS09195	58 -- 70	RsaE	58 -- 69	-11.82120	30S ribosomal protein S10
0.0281694	0.88564	SERP_RS02045	54 -- 67	RsaE	58 -- 69	-11.77880	DNA helicase RecQ
0.0296911	0.88564	SERP_RS04975	37 -- 45	RsaE	58 -- 66	-11.63960	YozE family protein

0.0299675	0.88564	SERP_RS10395	63 -- 71	RsaE	57 -- 65	-11.61510	L-serine ammonia-lyase iron-sulfur-dependent subunit alpha
0.0301974	0.88564	SERP_RS10190	69 -- 81	RsaE	53 -- 67	-11.59490	acetylornithine deacetylase
0.0303429	0.88564	SERP_RS11820	105 -- 114	RsaE	60 -- 69	-11.58220	hexose phosphate transporter
0.0309402	0.88564	SERP_RS07290	68 -- 78	RsaE	56 -- 66	-11.53070	DNA ligase (NAD(+)) LigA
0.0313316	0.88564	SERP_RS09080	59 -- 66	RsaE	58 -- 65	-11.49750	translation initiation factor IF-1
0.0320591	0.88564	SERP_RS11040	60 -- 67	RsaE	58 -- 65	-11.43690	Derived by automated computational analysis using gene prediction method: Protein Homology.
0.0321041	0.88564	SERP_RS10005	61 -- 69	RsaE	58 -- 66	-11.43320	GtrA family protein
0.0323717	0.88564	SERP_RS07725	80 -- 87	RsaE	25 -- 32	-11.41130	Derived by automated computational analysis using gene prediction method: Protein Homology.
0.0324946	0.88564	SERP_RS11280	53 -- 65	RsaE	58 -- 70	-11.40130	biofilm operon icaADBC HTH-type negative transcriptional regulator IcaR
0.0327332	0.88564	SERP_RS08065	139 -- 149	RsaE	58 -- 67	-11.38200	antirestriction protein ArdA
0.0337594	0.88564	SERP_RS02535	59 -- 67	RsaE	57 -- 65	-11.30060	methionine ABC transporter substrate-binding protein
0.0338132	0.88564	SERP_RS08860	59 -- 70	RsaE	57 -- 68	-11.29640	chromosome partitioning protein ParA
0.0339224	0.88564	SERP_RS07560	9 -- 20	RsaE	53 -- 64	-11.28790	YeeE/YedE family protein
0.033925	0.88564	SERP_RS05340	60 -- 68	RsaE	57 -- 65	-11.28770	peptidase T
0.033961	0.88564	SERP_RS07465	63 -- 70	RsaE	58 -- 65	-11.28490	GntR family transcriptional regulator
0.0358543	0.88564	SERP_RS07875	121 -- 133	RsaE	55 -- 68	-11.14200	Derived by automated computational analysis using gene prediction method: Protein Homology.
0.0361704	0.88564	SERP_RS07155	32 -- 46	RsaE	55 -- 69	-11.11890	radical SAM/CxCxxxxC motif protein YfkAB
0.0363412	0.88564	SERP_RS11125	117 -- 129	RsaE	57 -- 68	-11.10650	beta-class phenol-soluble modulin
0.0369118	0.88564	SERP_RS11455	65 -- 73	RsaE	58 -- 66	-11.06550	Derived by automated computational analysis using gene prediction method: Protein Homology.
0.0377506	0.88564	SERP_RS05425	11 -- 20	RsaE	53 -- 62	-11.00640	acetylglutamate kinase
0.0380663	0.88564	SERP_RS09605	61 -- 70	RsaE	57 -- 66	-10.98450	formimidoylglutamase
0.0403839	0.88564	SERP_RS07790	46 -- 64	RsaE	55 -- 70	-10.82920	addiction module antitoxin
0.0411399	0.88564	SERP_RS10360	64 -- 70	RsaE	54 -- 60	-10.78050	aldehyde dehydrogenase
0.0413378	0.88564	SERP_RS07250	65 -- 78	RsaE	56 -- 69	-10.76790	Derived by automated computational analysis using gene prediction method: Protein Homology.
0.0414024	0.88564	SERP_RS01470	25 -- 36	RsaE	58 -- 68	-10.76380	zinc-dependent alcohol dehydrogenase

0.0417175	0.88564	SERP_RS03085	56 -- 69	RsaE	58 -- 70	-10.74390	similar to 2'-5' RNA ligase Derived by automated computational analysis using gene prediction method: Protein Homology.
0.0419119	0.88564	SERP_RS02590	124 -- 133	RsaE	59 -- 68	-10.73170	cystatin-like fold lipoprotein
0.0420543	0.88564	SERP_RS04900	65 -- 72	RsaE	53 -- 60	-10.72280	MoxR family ATPase
0.0420607	0.88564	SERP_RS05525	111 -- 119	RsaE	60 -- 68	-10.72240	Derived by automated computational analysis using gene prediction method: Protein Homology.
0.0424455	0.88564	SERP_RS00860	132 -- 144	RsaE	53 -- 66	-10.69850	hypoxanthine-guanine phosphoribosyltransferase
0.0426564	0.88564	SERP_RS01460	53 -- 79	RsaE	55 -- 69	-10.68550	TIGR00268 family protein
0.0428912	0.88564	SERP_RS01420	60 -- 67	RsaE	58 -- 65	-10.67110	ABC transporter substrate-binding protein
0.0430632	0.88564	SERP_RS03310	121 -- 148	RsaE	55 -- 68	-10.66060	5-(carboxyamino)imidazole ribonucleotide synthase
0.0441961	0.88564	SERP_RS10615	61 -- 85	RsaE	53 -- 67	-10.59250	polyphosphate--AMP phosphotransferase
0.044409	0.88564	SERP_RS00880	83 -- 101	RsaE	57 -- 69	-10.57990	dihydropteroate synthase
0.0446025	0.88564	SERP_RS02315	137 -- 148	RsaE	56 -- 67	-10.56850	Derived by automated computational analysis using gene prediction method: Protein Homology.
0.0451659	0.88564	SERP_RS02410	58 -- 70	RsaE	57 -- 68	-10.53560	Derived by automated computational analysis using gene prediction method: Protein Homology.
0.0454598	0.88564	SERP_RS08605	63 -- 71	RsaE	57 -- 65	-10.51860	serine hydroxymethyltransferase
0.046433	0.88564	SERP_RS10565	33 -- 69	RsaE	25 -- 67	-10.46310	L-glutamate gamma-semialdehyde dehydrogenase
0.0464347	0.88564	SERP_RS01410	54 -- 68	RsaE	56 -- 70	-10.46300	aldo/keto reductase
0.0472213	0.88564	SERP_RS01870	62 -- 73	RsaE	59 -- 70	-10.41900	malate dehydrogenase
0.0480967	0.88564	SERP_RS13485	64 -- 71	RsaE	25 -- 32	-10.37090	formate dehydrogenase subunit delta
0.0481297	0.88564	SERP_RS02495	56 -- 64	RsaE	60 -- 68	-10.36910	nitroreductase
0.0489698	0.88564	SERP_RS11970	76 -- 86	RsaE	48 -- 58	-10.32380	MFS transporter
0.0492813	0.88564	SERP_RS06185	58 -- 67	RsaE	60 -- 69	-10.30720	aldehyde dehydrogenase
0.0503838	0.88564	SERP_RS12190	62 -- 71	RsaE	56 -- 65	-10.24930	sensor histidine kinase KdpD
0.0504686	0.88564	SERP_RS08660	56 -- 67	RsaE	58 -- 69	-10.24490	UDP-N-acetylglucosamine 1-carboxyvinyltransferase
0.0506348	0.88564	SERP_RS07210	58 -- 71	RsaE	53 -- 67	-10.23630	methionine aminopeptidase
