# Supplementary material for: The small non-coding RNA RsaE influences extracellular matrix composition in Staphylococcus epidermidis biofilm communities
Source: PLoS Pathog. 2019 Mar 14;15(3):e1007618. doi: 10.1371/journal.ppat.1007618 (PMC6435200; doi:10.1371/journal.ppat.1007618)
Supplement: S1 Text — Methods. (DOCX) [file ppat.1007618.s013.docx]

*Supporting information* for manuscript:

**The small non-coding RNA RsaE influences extracellular matrix composition in *Staphylococcus epidermidis* biofilm communities**

*Short title:* RsaE effects in *S. epidermidis* biofilms

Sonja M.K. Schoenfelder^1^, Claudia Lange^1^, Srinivasa Abishek Prakash, Gabriella Marincola, Maike F. Lerch, Freya D. R. Wencker, Konrad U. Förstner^2^, Cynthia M. Sharma, and Wilma Ziebuhr*

University of Würzburg, Institute of Molecular Infection Biology, Josef-Schneider-Str. 2, D-97080 Würzburg, Germany

^1^ These authors contributed equally to the work.

^2^ Present address: TH Köln, Faculty of Information Science and Communication Studies, ZB MED – Information Centre for Life Sciences, 50678 Cologne, Germany

**Correspondence to:*

Dr Wilma Ziebuhr

University of Würzburg

Institute of Molecular Infection Biology

Josef-Schneider-Str. 2

D-97080 Würzburg

Germany

Phone: 0049 (0)931 3182154

Fax: 0049 (0)931 3182578

Email: w.ziebuhr@mail.uni-wuerzburg.de

**Supporting information**

**Methods**

**Differential RNA sequencing (dRNA-seq).** Total RNA was extracted at early exponential phase (OD_600_ = 1) and remaining genomic DNA was eliminated by DNase I treatment. For depletion of processed transcripts, equal amounts of RNA were incubated with Terminator 5'-phosphate-dependent exonuclease (TEX) (Epicentre #TER51020) as previously described [1]. The transcripts were not fragmented in order to get mainly sequencing reads of the 5'-end of the transcripts. Libraries for Illumina sequencing of cDNA were constructed by vertis Biotechnology AG, Germany (http://www.vertis-biotech.com/), as described previously for eukaryotic microRNAs [2] but omitting the RNA size-fractionation step prior to cDNA synthesis. Equal amounts of RNA samples were poly(A)-tailed using poly(A) polymerase. Then, the 5'-triphosphates were removed by applying tobacco acid pyrophosphatase (TAP) resulting in 5'-monophosphate. Afterwards, a RNA adapter was ligated to the 5'-phosphate of the RNA. First-strand cDNA was synthesized by an oligo(dT)-adapter primer and the M-MLV reverse transcriptase. In a PCR-based amplification step using a high fidelity DNA polymerase the cDNA concentration was increased to 20-30 ng/µl. A library-specific barcode for multiplex sequencing was part of a 3'-sequencing adapter. The following adapter sequences flank the cDNA inserts:

TrueSeq Sense primer: 5'AATGATACGGCGACCACCGAGATCTACACTCTTTCCCTACACGACGCTCTTCCGATCT-3'

TrueSeq Antisense NNNNNN primer:

(NNNNNN = 6n barcode for multiplexing) 5'-CAAGCAGAAGACGGCATACGAGAT-NNNNNN GTGACTGGAGTTCAGACGTGTGCTCTTCCGATC(dT25)-3'

The resulting cDNA libraries were sequenced using a HiSeq 2500 machine (Illumina) in single-read mode and running 100 cycles. The RNA-seq data discussed in this publication have been deposited in NCBI's Gene Expression Omnibus [3] and are accessible through GEO Series accession number GSE126097 (https://www.ncbi.nlm.nih.gov/geo/query/acc.cgi?acc=GSE126097)

**RNA-seq data analysis.** In order to assure a high sequence quality, the Illumina reads in FASTQ format were trimmed with a cut-off phred score of 20 by the program fastq_quality_trimmer from FASTX toolkit version 0.0.13 (http://hannonlab.cshl.edu/fastx_toolkit/). The following steps were performed using the subcommand "create", "align" and "coverage" of the tool READemption [4] version 0.4.3 with default parameters. The poly(A)-tail sequences were removed and a size filtering step was applied in which sequences shorter than 12 nt were eliminated. The collections of remaining reads were mapped to the reference genome sequences (RefSeq accession NC_002976.3 and NC_006663.1) using segemehl version 0.2.0 [5]. Coverage plots in wiggle format representing the number of aligned reads per nucleotide were generated based on the aligned reads and visualized in the Integrated Genome Browser [6]. Each graph was normalized to the total number of reads that could be aligned from the respective library. To restore the original data range and prevent rounding of small error to zero by genome browsers, each graph was then multiplied by the minimum number of mapped reads calculated over all libraries. For the gene expression quantification annotation files in GFF3 format (same accession numbers as mentioned above for the reference sequences in FASTA format) were retrieved from NCBI. The number of reads that were overlapping with genome features in the TEX untreated libraries were quantified. The pairwise expression comparison based on these gene quantifications with READemption's subcommand "deseq" relies on DESeq2 version 1.18.1. Genes with a fold-change of equal or higher than 2.0 and a p-value below 0.05 were considered as differentially expressed. Functional enrichment analysis was performed with clusterProfiler [7]. Furthermore, differentially expressed genes were highlighted in a representation of the biochemical pathways using a custom made Python script and iPath [8]. A compilation of Unix shell, Python and R scripts that documents the complete data processing and analysis is accessible at Zenodo (https://www.ncbi.nlm.nih.gov/geo/query/acc.cgi?acc=GSE126097).

**Construction of inducible RsaE expression vectors.** The *rsaE* gene was PCR amplified from *S. epidermidis* PS2 genomic DNA using the primers listed in S2 Table (*i.e.* RsaE_BglII_for and RsaE_XmaI_rev). By employing restriction enzymes *Xma*I and *Bgl*II, the PCR product and pCG248 vector [9] were digested and subsequently ligated to create pCG248_*rsaE*_XmaI-BglII. The exact transcription start site from promoter p_xyl/tet_ on plasmid pCG248 was then determined by 5'-RACE, revealing transcription to begin 46 nucleotides upstream of the *rsaE* insert. To achieve transcription of *rsaE* with a correct length, the additional nucleotides were removed from the vector by using primers pCG248_out_5’P and rsaE_out_5’P (carrying 5'-phosphorylated ends; S2 Table), in an outward PCR followed by transformation in *E. coli* DC10B cells to generate plasmid pCG248_*rsaE*. The empty vector pCG248 was treated in the same manner using primers pCG248_out_5’P and pCG248_out2_5’P (S2 Table). The constructs (pCG248_empty and pCG248_*rsaE*) were then transformed into *S. aureus* PS187∆*hsdR*∆*sauUSI* cells by electroporation. This strain is deficient in type IV and type I restriction systems and host for phage Φ187. The constructs were then introduced into *S. epidermidis* cells via phage transduction (see [10] for protocol). Successful transduction was verified by sequencing with primers pRAB11-MCS_for and pCG248-MCS_rev (S2 Table). Clones were further identified as *S. epidermidis* via API®Staph (Biomérieux) test and as biofilm positive by colony morphology on Congo red agar (CRA, [11]).

**Construction of fluorescent reporter gene vectors.** To create plasmid p_(P*_rsaE_cfp*) the promoter region of *rsaE* was amplified from chromosomal DNA of *S. epidermidis* PS10 with primers RsaE_BamHI and RsaE_BglII (S2 Table). In parallel, primers cerul_BamHI and cerul_BglII_sd were used for an outward directed PCR with vector pCerulean (S2 Table, [12]) as template resulting in a linearized vector backbone carrying a promoterless *cfp* gene (blue fluorescence protein Cerulean, codon adapted for *Staphylococcus*), but retaining the *cfp* Shine Dalgarno (SD) sequence. Both PCR fragments were digested with restriction enzymes *Bam*HI and *Bgl*II, ligated with T4 DNA ligase and transformed in *E. coli* DC10B cells. Positive clones were transformed via electroporation into *S. epidermidis* cells. Successful transformation was verified by sequencing with primer pCerul_MCS_F and MF8 (S2 Table). Clones were further identified as *S. epidermidis* via API®Staph (Biomérieux) test and as biofilm positive by colony morphology on CRA. For construction of plasmid p_(P*_cidA_cfp*/P_lrgA_*yfp*), the promoter region of gene *cidA* including its SD sequence was amplified by PCR with primers cidA_BamHI and cidA_BglII (S2 Table) from chromosomal DNA of *S. epidermidis*. Vector pCerulean was again linearized with outward directed primers cerul_BamHI and cerul_BglII resulting in a vector backbone harbouring the *cfp* gene without promoter and SD sequence. After restriction enzyme digest (*Bam*HI and *Bgl*II), T4 ligation and transformation in *E. coli* DC10B cells, positive clones carrying p_(P*_cidA_cfp*) were re-isolated and subjected to further restriction digest (*Sal*I and *Bam*HI). In parallel, the promoter region of gene *lrgA* (including its SD sequence) was amplified by PCR with primers lrgA_SalI and lrgA_BamHI_HindIII (S2 Table) and cloned into the pGEM®-T Easy Vector System I (Promega). The restriction sites *Hind*III and *Bam*HI (introduced with the primer) were then used to insert the *yfp* gene (yellow fluorescent protein) which was yielded from vector pKM003 using the same restriction enzymes (Table 1) [13]. The complete P_l_*_rgA_yfp* fragment was then released from the pGEM®-T Easy Vector by restriction enzymes *Sal*I and *Bam*HI and ligated with vector p_(P*_cidA_cfp)* (see above). The double promoter fusion construct was then transformed via electroporation into *S. epidermidis* cells. Successful transformation was ensured by sequencing with primers pCerul_MCS_F, pCerul_MCS_R and MF8 (S2 Table). Clones were further identified as *S. epidermidis* via API®Staph (Biomérieux) test and as biofilm positive by colony morphology on CRA.

**Preparation of *in vitro* transcription (IVT) templates.** DNA templates for *in vitro* transcription (IVT) of RNAs used in EMSAs were generated by PCR from genomic DNA of *S. epidermidis* PS2 with the primer pairs listed in S2 Table. For mutation of the putative interaction site of *lrgA*, the PCR product of *lrgA* was first transformed into the pGEM®-T Easy Vector System I (Promega) resulting in pGEM_*lrgA* and then subjected to site directed mutagenesis (SDM) PCR with outward directed primers For_LrgA_SDM and Rev_LrgA_SDM (S2 Table) containing the desired 6 bp nucleotide exchanges, thereby creating plasmid pGEM_mut*lrgA*. For IVT template generation of mut*lrgA* the same primers as for *lrgA* were used (S2 Table), but with plasmid pGEM_mut*lrgA* instead of genomic DNA as template. Mutation of the ribosomal binding site (RBS) in 5’-UTR-*icaR* was done in the same way, by first using primers icaR5’_SDG4Umut_1 and icaR5’_SDG4Umut_2 (S2 Table) to exchange the 4 G’s to 4 U’s by SDM and then primers F_icaR5'_T7 and R_icaR5’ (S2 Table) on the mutated DNA to create the template 5’-UTR-*icaR*-RBSmut for IVT.

**Quantitative reverse transcription PCR (qRT–PCR).** For qRT–PCR, 5 μg of each RNA sample was treated with DNaseI (Roche, Mannheim, Germany) for 30 min at room temperature. One-step qRT–PCR was performed using an amplification kit with SYBR Green I (Power SYBR™ Green RNA-to-CT™ 1-Step Kit; Thermofisher) with the specific primers listed in Supporting Information Table S2 and run on Biorad CFX according to the manufacturer’s instructions (Biorad). The RNA was diluted (1:10) and transcript abundance was calculated using a logarithmic dilution series of one sample to generate a standard curve for each gene. Relative quantification of the genes of interest was expressed in relation to the expression of the constitutive reference gene gyrase (*gyrB*). The means were calculated from three biological replicates run in duplicate.

**Construction of markerless deletion mutant strain of *rsaE* and inducible *rsaE* expression strains.** The flanking region of *rsaE* was PCR amplified using primers F_delRsaE_SalI and R_delRsaE_SalI (S2 table). The PCR product and pBASE6 vector [14] were digested using SalI restriction enzyme and subsequently ligated. Primers RsaE_out1_5'p and RsaE_out2_5'p (S2 table) were used with this plasmid as a template for an outward directed PCR for *rsaE* deletion from this construct to create pBASE6_flanking region_*rsaE*. This plasmid was introduced into *S. epidermidis* PS10 by phage Φ187 mediated transduction and the double crossover protocol for vector pBASE6 was used for *rsaE* deletion [15]. Successful deletion of *rsaE* was confirmed by PCR using primers SAP-rsaE-del2-F and SAP-rsaE-del2-R (S2 table). For complementation of the *rsaE* deletion mutant by ATc controlled induction of *rsaE* transcription, the plasmid pCG248_*rsaE* was transduced into *S. epidermidis* PS10∆*rsaE* using phage Φ187. Successful transduction was verified by sequencing with primers pRAB11-MCS_for and pCG248-MCS_rev2 (S2 table).

**Complementation of *rsaE* deletion mutant with a mutated version of *rsaE***. The plasmid pCG248_*rsaE* was used as the template for the mutations. Motifs 1, 2 and 3 (S4 figure) of *rsaE* were mutated by exchanging the C’s to A’s by outward directed PCR using three primer sets. RsaE_mot1C4A_out1 and RsaE_mot1C4A_out2 (S2 table) was used for motif 1 mutation, SAP_rsaE mot2 mut C4A for and SAP_rsaE mot2 mut C4A rev (S2 table) was used for motif 2 mutation and RsaEtrunc_mot3C4A_out1 and RsaEtrunc_mot3C4A_out2 (S2 table) was used for motif 3 mutation. Successful mutations were verified by sequencing with primers pRAB11-MCS_for and pCG248-MCS_rev2. The resulting plasmids were introduced into *S. epidermidis* PS10∆*rsaE* by phage Φ187 mediated transduction.

**Interactive visualization of the RNAseq data set.** For visualization and interactive analysis of the transcriptome data (including regulatory pathways and secondary metabolite biosynthesis genes) go to the 'Interactive Pathways Explorer v3' at <https://pathways.embl.de>/ [8]. Select a map of interest (*i.e.* metabolism or secondary metabolites), copy the data set provided below into the 'Element selection' window of the customize area of the programme and submit data. The data set contains differentially expressed genes of *S. epidermidis* PS2 compared to PS10 with a minimum log2-fold-change of 1 and a p value ≤0.05.

Data set for upload into Interactive Pathway Explorer:

K03436 #7ADEFF W15

nan #7ADEFF W15

K10254 #7ADEFF W15

K20337 #7ADEFF W15

K20337 #7ADEFF W15

K20337 #7ADEFF W15

nan #7ADEFF W15

K00164 #7ADEFF W15

K00134 #7ADEFF W15

K00318 #7ADEFF W15

K01610 #7ADEFF W15

nan #7ADEFF W15

nan #7ADEFF W15

K01318 #7ADEFF W15

K01990 #7ADEFF W15

K11039 #7ADEFF W15

K07800 #7ADEFF W15

K07706 #7ADEFF W15

K07707 #7ADEFF W15

nan #7ADEFF W15

K01220 #7ADEFF W15

K02787 #7ADEFF W15

K02788 #7ADEFF W15

K02786 #7ADEFF W15

K01635 #7ADEFF W15

K00917 #7ADEFF W15

K01819 #7ADEFF W15

K01819 #7ADEFF W15

K02749 #7ADEFF W15

K02750 #7ADEFF W15

K05338 #7ADEFF W15

K05339 #7ADEFF W15

K03535 #7ADEFF W15

K00128 #7ADEFF W15

K06216 #7ADEFF W15

K00294 #7ADEFF W15

K09773 #7ADEFF W15

K01006 #7ADEFF W15

K01478 #7ADEFF W15

K01809 #7ADEFF W15

K00627 #7ADEFF W15

K21417 #7ADEFF W15

K21416 #7ADEFF W15

K00382 #7ADEFF W15

K00611 #7ADEFF W15

K01439 #7ADEFF W15

K08258 #7ADEFF W15

K17217 #FF99AD W15

K02437 #FF99AD W15

K01923 #FF99AD W15

K03671 #FF99AD W15

K02777 #FF99AD W15

nan #FF99AD W15

K01104 #FF99AD W15

K01839 #FF99AD W15

K09813 #FF99AD W15

K02424 #FF99AD W15

K00860 #FF99AD W15

K00958 #FF99AD W15

K07146 #FF99AD W15

**References**

1. Sharma CM, Hoffmann S, Darfeuille F, Reignier J, Findeiss S, Sittka A, et al. The primary transcriptome of the major human pathogen *Helicobacter pylori*. Nature. 2010;464(7286):250-5. doi: 10.1038/nature08756. PubMed PMID: 20164839.

2. Berezikov E, Cuppen E, Plasterk RH. Approaches to microRNA discovery. Nat Genet. 2006;38 Suppl:S2-7. doi: 10.1038/ng1794. PubMed PMID: 16736019.

3. Edgar R, Domrachev M, Lash AE. Gene Expression Omnibus: NCBI gene expression and hybridization array data repository. Nucleic Acids Res. 2002;30(1):207-10. PubMed PMID: 11752295; PubMed Central PMCID: PMCPMC99122.

4. Forstner KU, Vogel J, Sharma CM. READemption-a tool for the computational analysis of deep-sequencing-based transcriptome data. Bioinformatics. 2014;30(23):3421-3. doi: 10.1093/bioinformatics/btu533. PubMed PMID: 25123900.

5. Hoffmann S, Otto C, Kurtz S, Sharma CM, Khaitovich P, Vogel J, et al. Fast mapping of short sequences with mismatches, insertions and deletions using index structures. PLoS Comput Biol. 2009;5(9):e1000502. doi: 10.1371/journal.pcbi.1000502. PubMed PMID: 19750212; PubMed Central PMCID: PMCPMC2730575.

6. Freese NH, Norris DC, Loraine AE. Integrated genome browser: visual analytics platform for genomics. Bioinformatics. 2016;32(14):2089-95. doi: 10.1093/bioinformatics/btw069. PubMed PMID: 27153568; PubMed Central PMCID: PMCPMC4937187.

7. Yu G, Wang LG, Han Y, He QY. clusterProfiler: an R package for comparing biological themes among gene clusters. OMICS. 2012;16(5):284-7. doi: 10.1089/omi.2011.0118. PubMed PMID: 22455463; PubMed Central PMCID: PMCPMC3339379.

8. Yamada T, Letunic I, Okuda S, Kanehisa M, Bork P. iPath2.0: interactive pathway explorer. Nucleic Acids Res. 2011;39(Web Server issue):W412-5. doi: 10.1093/nar/gkr313. PubMed PMID: 21546551; PubMed Central PMCID: PMC3125749.

9. Helle L, Kull M, Mayer S, Marincola G, Zelder ME, Goerke C, et al. Vectors for improved Tet repressor-dependent gradual gene induction or silencing in Staphylococcus aureus. Microbiology. 2011;157(Pt 12):3314-23. doi: 10.1099/mic.0.052548-0. PubMed PMID: 21921101.

10. Winstel V, Kuhner P, Rohde H, Peschel A. Genetic engineering of untransformable coagulase-negative staphylococcal pathogens. Nat Protoc. 2016;11(5):949-59. doi: 10.1038/nprot.2016.058. PubMed PMID: 27101516.

11. Freeman DJ, Falkiner FR, Keane CT. New method for detecting slime production by coagulase negative staphylococci. J Clin Pathol. 1989;42(8):872-4. PubMed PMID: 2475530; PubMed Central PMCID: PMCPMC1142068.

12. Paprotka K, Giese B, Fraunholz MJ. Codon-improved fluorescent proteins in investigation of *Staphylococcus aureus* host pathogen interactions. J Microbiol Methods. 2010;83(1):82-6. doi: 10.1016/j.mimet.2010.07.022. PubMed PMID: 20708040.

13. Sullivan NL, Marquis KA, Rudner DZ. Recruitment of SMC by ParB-parS organizes the origin region and promotes efficient chromosome segregation. Cell. 2009;137(4):697-707. doi: 10.1016/j.cell.2009.04.044. PubMed PMID: 19450517; PubMed Central PMCID: PMCPMC2892783.

14. Geiger T, Francois P, Liebeke M, Fraunholz M, Goerke C, Krismer B, et al. The stringent response of *Staphylococcus aureus* and its impact on survival after phagocytosis through the induction of intracellular PSMs expression. PLoS Pathog. 2012;8(11):e1003016. Epub 2012/12/05. doi: 10.1371/journal.ppat.1003016 PPATHOGENS-D-12-01227 [pii]. PubMed PMID: 23209405; PubMed Central PMCID: PMC3510239.

15. Bae T, Schneewind O. Allelic replacement in *Staphylococcus aureus* with inducible counter-selection. Plasmid. 2006;55(1):58-63. doi: 10.1016/j.plasmid.2005.05.005. PubMed PMID: 16051359.
